# Alterporriol-Type Dimers from the Mangrove Endophytic Fungus, *Alternaria* sp. (SK11), and Their MptpB Inhibitions

**DOI:** 10.3390/md12052953

**Published:** 2014-05-16

**Authors:** Guoping Xia, Jia Li, Hanxiang Li, Yuhua Long, Shao’e Lin, Yongjun Lu, Lei He, Yongcheng Lin, Lan Liu, Zhigang She

**Affiliations:** 1 School of Chemistry and Chemical Engineering, Sun Yat-Sen University, Guangzhou 510275, China; E-Mails: xiagp@mail2.sysu.edu.cn (G.X.); nuekagami@163.com (J.L.); lihx3@mail.sysu.edu.cn (H.L.); linshaoe@mail2.sysu.edu.cn (S.L.); ceslyc@mail.sysu.edu.cn (Y.L.); 2 Department of Pharmacy, Huaihua Medical College, Huaihua 418000, China; 3 School of Chemistry and Environment, South China Normal University, Guangzhou 510006, China; E-Mail: longyh@scnu.edu.cn; 4 School of Life Sciences, Sun Yat-Sen University, Guangzhou 510275, China; E-Mails: luyj@mail.sysu.edu.cn (Y.L.); helei8688@126.com (L.H.)

**Keywords:** endophytic fungus, *Alternaria* sp., alterporriol, theoretical calculations, ECD, TDDFT, MptpB

## Abstract

A new alterporriol-type anthranoid dimer, alterporriol S (**1**), along with seven known anthraquinone derivatives, (+)-a*S*-alterporriol C (**2**), hydroxybostrycin (**3**), halorosellinia A (**4**), tetrahydrobostrycin (**5**), 9α-hydroxydihydrodesoxybostrycin (**6**), austrocortinin (**7**) and 6-methylquinizarin (**8**), were isolated from the culture broth of the mangrove fungus, *Alternaria* sp. (SK11), from the South China Sea. Their structures and the relative configurations were elucidated using comprehensive spectroscopic methods, including 1D and 2D NMR spectra. The absolute configurations of **1** and the axial configuration of **2** were defined by experimental and theoretical ECD spectroscopy. **1** was identified as the first member of alterporriols consisting of a unique C-10−C-2′ linkage. Atropisomer **2** exhibited strong inhibitory activity against *Mycobacterium* *tuberculosis* protein tyrosine phosphatase B (MptpB) with an IC_50_ value 8.70 μM.

## 1. Introduction

Tuberculosis (TB) is one of the greatest killers, responsible for 8.6 million infections and 1.3 million deaths in 2012, according to the WHO [1]. *Mycobacterium tuberculosis* is the causative agent of TB and the deserved target of antituberculosis drugs. In recent years, extensively drug-resistant TB, multidrug-resistant TB and HIV-associated TB have made clinical treatment even more difficult and complex. Novel anti-infective agents are in urgent need, especially those applying to new targets and based on different mechanisms. *Mycobacterium* *tuberculosis* protein tyrosine phosphatase B (MptpB) is proven to be an essential virulence factor when *M. tuberculosis* hosts macrophages [2,3]. Increased research reveals that it exhibits unique and multiple activities against immune responses [4,5,6,7]. Therefore, developing selective MptpB inhibitors could be a promising strategy against *M. tuberculosis* infection and conducive to treating severe TB.

**Scheme 1:**
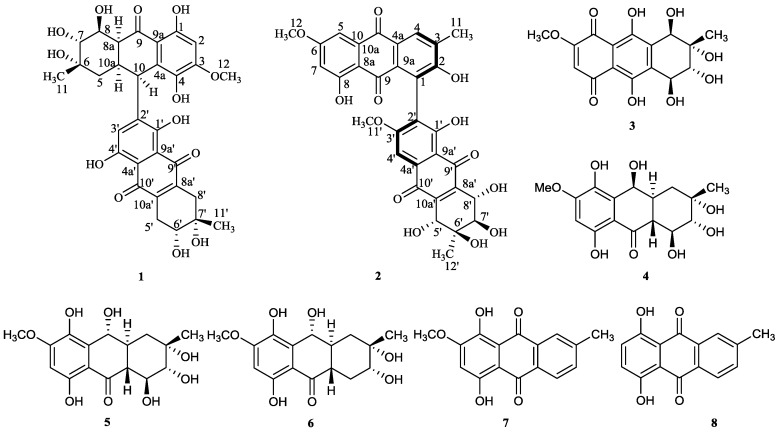
Structures of **1**–**8** isolated from *Alternaria* sp. (SK11).

As part of our ongoing investigation on natural antituberculosis products from marine fungi in the South China Sea [8,9,10], a mangrove endophytic fungus, *Alternaria* sp. (SK11), attracted our attention for 4-deoxybostrycin, a natural anthraquinone compound isolated from this strain, showing good inhibition against some clinical multidrug-resistant *M. tuberculosis* strains [8]. Further chemical investigation of this fungus led to the isolation of eight metabolites (Scheme 1), including one novel alterporriol-type anthranoid dimer, alterporriol S (**1**), and seven known compounds, (+)-a*S*-alterporriol C (**2**), hydroxybostrycin (**3**), halorosellinia A (**4**), tetrahydrobostrycin (**5**), 9α-hydroxydihydrodesoxybostrycin (**6**), austrocortinin (**7**) and 6-methylquinizarin (**8**). In this report, we describe the isolation, structural elucidation and biological activity of the metabolites.

## 2. Results and Discussion

The marine-derived fungus SK11 was identified as *Alternaria* sp. on the basis of molecular characteristics combined with morphological traits. All compounds were isolated using chromatographic techniques, and their structures were elucidated by spectroscopic data (IR, UV, NMR) and HRMS. Their relative configurations were assigned according to 1D NMR and NOESY experiments. The absolute charities were established by the electronic circular dichroism (ECD) method supported by the time-dependent density functional theory (TDDFT) calculations of ECD spectra.

**Figure 1:**
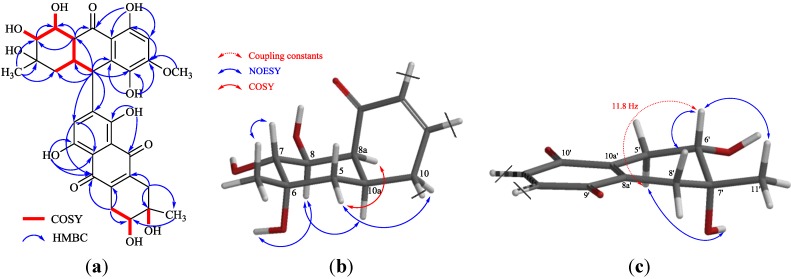
^1^H–^1^H COSY, key HMBC (**a**) and meaningful NOESY; (**b**,**c**) correlations of **1**.

Compound **1**, with the molecular formula C_31_H_32_O_13_ from HRESIMS data (*m*/*z* 611.1793 [M − H]^ −^), was obtained as a red, amorphous powder. The presence of UV absorption bands at 362.2, 288.8, 248.6 and 220.4 nm indicated the existence of a conjugated carbonyl chromophore [11]. Furthermore, a hydroxy absorption band was found at 3432 cm^−1^, while carbonyl ones were found at 1653 cm^−1^, in the IR spectrum. The presence of three chelated hydroxy proton signals (δ_H_ 13.17, 12.46, 12.42), five changeable hydroxy proton signals (δ_H_ 4.82, 4.62, 4.49, 4.33, 4.06), two methyls (δ_H_ 1.18, 1.08), two aromatic protons (δ_H_ 6.73, 6.34) in ^1^H NMR and three carbonyl carbon signals (δ_C_ 206.4, 181.9, 180.9) in ^13^C NMR (Table 1) suggested that this compound could be a tetrahydroanthraquinone heterodimer. In the ^1^H NMR spectrum, signals corresponding to the northern moiety contained two doublets at δ_H_ 4.62 and δ_H_ 4.33 and a singlet at δ_H_ 4.06, assigned to 7-OH, 8-OH and 6-OH, respectively, as well as a singlet that corresponded to the methyl group, 6-Me, resonating at δ_H_ 1.08. In addition, an isolated proton (2-H) and a methoxy group (3-OMe) were detected at δ_H_ 6.73 and δ_H_ 3.89, respectively. The ^1^H−^1^H COSY spectrum of **1** revealed that two oxygenated methine groups at δ_H_ 3.20 (7-H) and 3.45 (8-H), three methine groups at δ_H_ 2.99 (8a-H), 2.64 (10a-H) and 4.60 (10-H) and one methylene group at δ_H_ 1.65 (5-H_eq_) and 1.37 (5-H_ax_) allowed an aliphatic spin system, ^7^CHO−^8^CHO−^8a^CH−^10a^CH(−^10^CH)−^5^CH_2_ (Figure 1a). Furthermore, in the HMBC spectrum, the correlations attributed to 5-H (5-H_eq_ and 5-H_ax_) with C-10, C-10a, 7-H with C-8, 8a-H with C-5, C-7, C-8, C-9 and C-10 and 10-H with C-4, C-4a, C-5, C-8a, C-9a and C-10a, as well as those of the isolated aromatic proton (2-H/C-1, C-3, C-4 and C-9a) fully supported the assignment of the planar structure of the northern moiety of the molecule. The observed HMBC correlations from 10-H to the aromatic carbons of the southern moiety, C-2′, C-3′ and C-4′, were vital to assign the position of the linkage. Moreover, in analogy to known Compounds **4** or **5**, the oxygenated methine group (δ_C_ 61.8 or δ_C_ 72.2) [12,13] was now replaced by a methine group at C-10 (δ_C_ 37.1) in **1**. The moderate upfield-shift of the 10-H signal (δ_H_ 4.73 in **4** or δ_H_ 4.91 in **5**→δ_H_ 4.60 in **1**) may be ascribed to the deshielding effect of the neighboring tetrahydroanthraquinone system [14,15], thus supporting the northern moiety attached to the aromatic ring of the other moiety via C-10. 2D NMR correlations were used to identify the structure of the southern moiety in **1**, as well. In the COSY spectrum, the low-field doublet (6′-OH) correlated with the high-field aliphatic proton 6′-H, which further correlated with 5′-H. Together with the observed HMBC correlations of 5′-H/C-6′, C-10′ and C-10a′, 6′-H/C-11′, and 8′-H/C-7′, C-8a′ and C-11′, the aliphatic ring was allowed to be established as shown (Figure 1a). Moreover, the aromatic proton observed at δ_H_ 6.34 was assigned to 3′-H by interpretation of the HMBC spectrum, which revealed correlations of the chelated hydroxy signal at δ_H_ 13.17 and δ_H_ 12.42 with carbonyl carbon C-9′ and C-10′, respectively. These data suggested that the southern moiety of **1** was demethoxyl 4-deoxybostrycin [12,16], and the link site in this half of the molecule was located at C-2′. Thus, **1** was deduced to be a new alterporriol-type dimer with a unique C-10−C-2′ linkage and given the name alterporriol S.

**Table 1:** NMR spectroscopic data (400 MHz, DMSO-*d*_6_) for **1** *^a^*.

Position	δ_C_	δ_H_	HMBC *^b^*
1	156.9, C		
2	99.5, CH	6.73, s	1, 3, 4, 9a
3	157.0, CH		
4	136.5, C		
4a	125.5,C		
5_ax_ 5_eq_	42.0, CH_2_	1.37, dd (12.8, 13.5); 1.65, dd (12.8, 3.8)	10, 10a
6	71.7, C		
7	75.6, CH	3.20, dd (10.0, 4.9)	8
8	73.1, CH	3.45, ddd (10.0, 4.9, 4.6)	7
8a	46.0, CH	2.99, ddd (4.6, 4.1, 1.2)	5, 7, 8, 9, 10
9	206.4, C		
9a	110.2, C		
10	37.1, C	4.60, brs	4, 4a, 5, 8a, 9a, 10a, 2′, 3′, 4′
10a	35.1, C	2.64, m	11
11	26.7, CH_3_	1.08, s	5, 6, 7
12	56.7, CH_3_	3.89, s	3
1-OH		12.46, s	1, 2, 9a
4-OH		8.57, s	3, 4, 4a
6-OH		4.06, s	
7-OH		4.62, d (4.9)	
8-OH		4.33, d (4.9)	
1′	161.8, C		
2′	146.3, C		
3′	127.6, CH	6.34, s	10, 1′, 4a′
4′	162.5, C		
4a′	110.5, C		
5′_ax_ 5′_eq_	30.2, CH_2_	2.65, m 2.80, dd (14.4, 5.0)	6′, 10′ *^c^*, 10a′
6′	70.6, CH	3.61, dt (11.8, 5.0)	11′ *^c^*
7′	69.4, C		
8′	35.9, CH_2_	2.74, d (14.1) 2.58, m	7′, 8a′, 11′
8a′	142.3, C		
9′	180.9, C		
9a′	111.5, C		
10′	181.9, C		
10a′	142.8,C		
11′	25.7, CH_3_	1.18, s	6′, 8′
1′-OH		13.17, s	1′, 2′, 9′
4′-OH		12.42, s	3′, 4′, 4a′,10′
6′-OH		4.82, d (5.0)	
7′-OH		4.49, s	

*^a^* δ in ppm, *J* in Hz, TMS as the internal standard; *^b^* HMBC correlations, optimized for 6 Hz, were started from the proton to the indicated carbon; *^c^* signal partially obscured.

The coupling constants observed in the ^1^H NMR spectrum, as well as the correlations detected in the NOESY spectrum were used to determine the relative configurations of Compound **1**. 5-H_ax_ and 10a-H were revealed to have a *trans*-*diaxial* relationship for the big coupling constant (*J* = 13.5 Hz) between the two protons. 7-H and 8-H were also placed in *trans-axial* positions for the large ^3^*J*_7-H–8-H_ value (10.0 Hz). 8a-H was placed on the same side of the aliphatic ring with 10a-H for the small coupling constant (*J* = 4.1 Hz) between the two protons, i.e., equatorial position, which explained the long-range correlation between 8a-H and 5-H_eq_ observed in the COSY spectrum (*J* = 1.2 Hz). The 10-H signal appeared as a broadened singlet without significant splitting in spite of the obvious COSY correlation between 10-H and 10a-H, which suggested that the coupling constant between them was considerably small; thus, 10-H was placed in a *cis* position with 10a-H. In the NOESY experiment, the correlation of 6-OH/8-H and 6-Me/7-H was observed, which allowed the equatorial position of 6-Me to be established (Figure 1b). Consequently, the relative configurations of the northern moiety in **1** were 6S*, 7R*, 8S*, 8aS*, 10R*, 10aR*. In the southern moiety, 6′-H was in an axial position according to the big coupling constant (*J* = 11.8 Hz) between 6′-H and 5′-H_ax_. 7′-OH was placed in an axial position for the correlations of 5′-H_ax_/7′-OH and 6′-H/7′-Me observed in the NOESY spectrum (Figure 1c). Thus, the relative configurations of the southern moiety in **1** were established as 6′R*, 7′S*.

**Figure 2:**
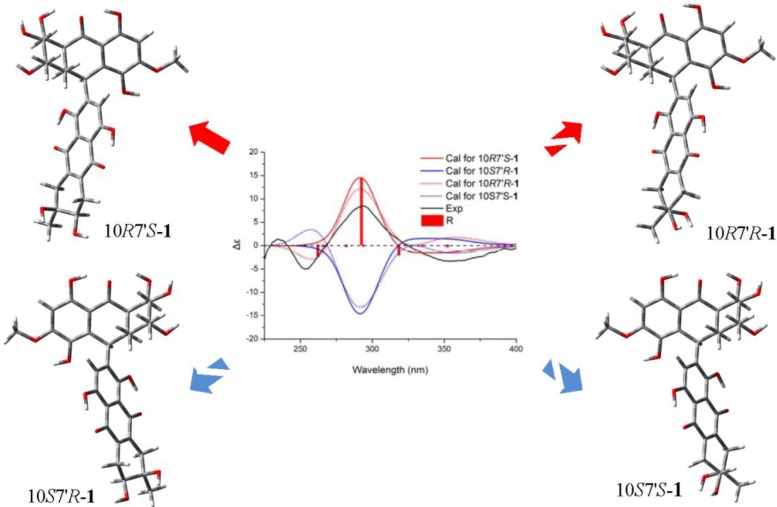
Experimental and calculated ECD spectra of **1** *.

* Black solid line: ECD spectrum of **1** in MeOH solution; red solid line: calculated ECD spectrum for 10*R*7′*S-***1** with the B3LYP/6-311G++(2d,p) method on the DFT-optimized structure; red dotted line: calculated ECD spectrum for 10*S*7′*R-***1** with the B3LYP/6-311G++(2d,p) method on the DFT-optimized structure; blue solid line: calculated ECD spectrum for 10*R*7′*R*-**1** with the B3LYP/6-311G++(2d,p) method on the DFT-optimized structure; blue dotted line: calculated ECD spectrum for 10*S*7′*S*-**1** with the B3LYP/6-311G++(2d,p) method on the DFT-optimized structure; red bars: rotational strengths of the lowest-energy conformer of 10*R*7′*S*-**1** optimized with B3LYP/6-31G(d). Spectra were generated with σ = 0.16 eV.

To assign the absolute configurations of **1**, the ECD spectrum was measured in methanol solution and compared with that calculated by quantum-mechanics. The resulting CD was substantially affected by eight chiral centers in the molecular as a consequence of the central C-10-C–2′ axis being able to rotate rather freely in solution, due to low rotational barriers [15]. As shown in Figure 2, several bands between 200 and 400 nm, due to the various π–π* and n–π* transitions of the substituted naphthoquinone chromophore, were observed in the experimental ECD spectrum. In particular, two couplet-like features appeared in the two regions between 225–265 and 275–400 nm. The conformational analyses of **1** were carried out with molecular mechanics (using the Merck molecular force field, MMFF) based on a Monte Carlo algorithm. The initial structures were built with the four possible diastereomers, which were obtained by using a standard procedure for flexible molecules [17]. For each configuration of **1**, four low-energy conformers were obtained and re-optimized with DFT at the B3LYP/6-31G(d) level. The various minima differed in the orientation of the aliphatic rings, methyl and hydroxy groups and aryl–aryl torsions, with energies that differed by less than 1 kcal/mol and amounted to >95% overall Boltzmann population at 300 K. The energies, oscillator strengths and rotational strengths of the first 10 electronic excitations were calculated using the TDDFT methodology at the B3LYP/6-31++G(2d,p) level [18], including the IEF-PCM solvent model for methanol. The simulated spectra of the three lowest energy conformations were averaged according to the Boltzmann distribution theory to get the final spectra [19]. Simulations of the 10*R* and 10*S* diastereomers provided significant resolution at *ca*. 290 nm, which allowed the assignment of the absolute configuration of the northern moiety in **1** as 6*S*, 7*R*, 8*S*, 8a*S*, 10*R*, 10a*R*. The difference in averaged CD spectra between *ca*. 315 and 400 nm was crucial to distinguish 7′*R* and 7′*S* diastereomers. By careful comparison of the stimulated CD spectra of every low-energy conformers (see Supplementary Charts S1 and S2), it is proven that all the stimulated CD spectra of low-energy conformers of 7′*S*-**1** showed negative CE (Cotton effect) in the region 315–400 nm. On the contrary, all those of the low-energy conformers of 7*R*′-**1** showed positive CE. Consequently, 7′*R* and 7′*S* diastereomers were distinguishable by comparing their CE in this region (Figure 2), and the absolute configurations of the southern moiety in **1** were 6′*R*, 7′*S*.

**Figure 3:**
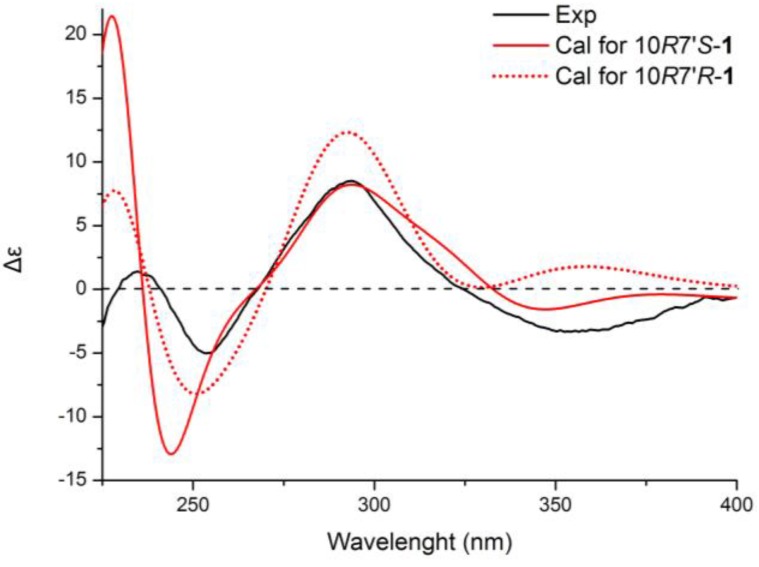
Calculated ECD spectra of 10*R*7′*S* and 10*R*7′*S* diastereomers.

The results were fully verified by recalculating with TDDFT at the CAM–B3LYP/6-31++G(2d,p) level. As shown in Figure 3, 7′R and 7′S diastereomers generated oppositely signed CE in the region of 315–400 nm, which were previously proven by the TDDFT B3LYP/6-31++G(2d,p) methodology. Moreover, the CAM–B3LYP functional gave better fitting with the experiment curve in 225–250 nm. Thus, it was safer to conclude that the absolute configurations of the southern moiety in **1** were 6′*R*, 7′*S*.

* Black solid line: ECD spectrum of **1** in MeOH solution; red solid line: calculated ECD spectrum for 10*R*7′*S-***1** with the CAM–B3LYP/6-311G++(2d,p) method on the DFT-optimized structure; red dotted line: calculated ECD spectrum for 10*S*7′*R-***1** with the CAM−B3LYP/6-311G++(2d,p) method on the DFT-optimized structure. Spectra were generated with σ = 0.20 eV.

**Figure 4:**
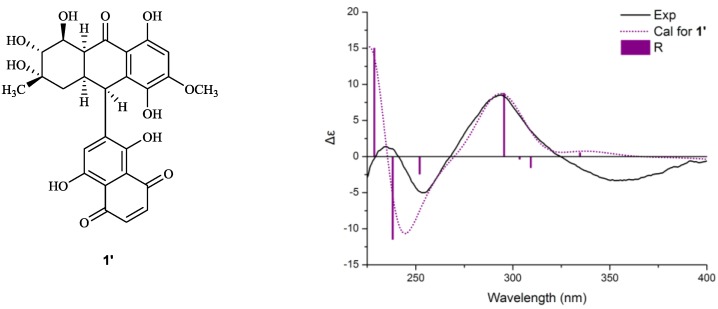
Calculated ECD spectra of **1**′.

Moreover, all the DFT optimized conformers of **1** were simplified by reducing the chiral aliphatic ring in the southern moiety. The energies, oscillator strengths and rotational strengths of the first 20 electronic excitations were then calculated using TDDFT methodology at the CAM-B3LYP/6-311++G(2d,p) level. The stimulated ECD spectrum of the hypothetical compound (**1**′) (Figure 4) showed a similar absorption curve with the experimental one with an exception in 315–400 nm, which fully supported that the first CE was generated by an asymmetric environment in the southern moiety of the molecular, and significant CE at *ca*. 290 nm was dominated by the chirality of the northern moiety. Thus, the absolute configurations of **1** were 6*S*, 7*R*, 8*S*, 8a*S*, 10*R*, 10a*R*, 6′*R* and 7′*S*.

* Black solid line: ECD spectrum of **1** in MeOH solution; purple dotted line: calculated ECD spectrum for **1′**; purple bars: rotational strengths of the lowest-energy conformer of 1′. Spectra were generated with σ = 0.16 eV.

**Figure 5:**
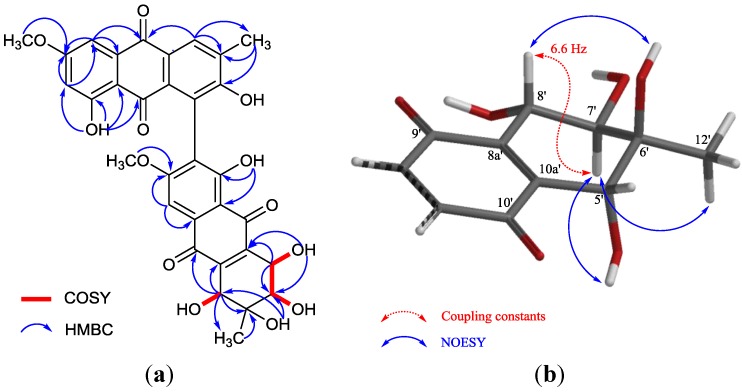
^1^H–^1^H COSY, key HMBC (**a**) and meaningful NOESY; (**b**) correlations of **2**.

Compound **2** was obtained as an orange, amorphous powder. By interpretation of HRESIMS, UV, IR and NMR spectra, the planar structure of **2** was fully assigned (Figure 5a), which was identical to alterporriol C, previously isolated from *A. porri* [20]. We report herein the NMR data recorded in DMSO-*d*_6_ (Table 2).

**Table 2:** NMR spectroscopic data (400 MHz, DMSO-*d*_6_) for **2** *^a^*.

Position	δ_C_	δ_H_	HMBC *^b^*
1	125.5, C		
2	159.1, C		
3	131.0, C		
4	129.7, CH	8.05, s	2, 3, 10 *^c^*, 11
4a	131.6, C		
5	106.6, CH	7.16, d (2.3)	6, 8a, 9, 10, 10a
6	165.6, C		
7	106.9, C	6.76, d (2.2)	5, 6, 8a, 9
8	164.5, C		
8a	110.3, C		
9	187.6, C		
9a	106.1, C		
10	180.8, C		
10a	134.5, C		
11	17.3, CH_3_	2.31, s	2, 3, 9
12	56.8, CH_3_	3.89, s	6
2-OH		9.50, brs	
8-OH		12.48, s	7, 8, 8a, 9
1′	163.9, C		
2′	126.0, C		
3′	164.2, C		
4′	104.1, CH	6.96, s	3′, 4a′, 9a′
4a′	123.0, C		
5′	68.4, CH	4.05, s	6′, 8a′, 10′,10a′, 12′
6′	73.0, C		
7′	68.2, CH	3.57, dd (5.6, 6.6)	8′
8′	73.8, CH	4.48, m	7′, 8a′, 10a′
8a′	142.3, C		
9′	189.1, C		
9a′	109.7, C		
10′	184.4, C		
10a′	143.6, C		
11′	56.3, CH_3_	3.71, s	3′
12′	22.3, CH_3_	1.12, s	5′, 6′
1′-OH		13.09, s	1′, 4′, 9a′
5′-OH		5.75, brs	
6′-OH		4.38, s	5′, 6′, 7′
7′-OH		4.86, d (5.6)	
8′-OH		5.05, brs	6′, 7′, 8a′

*^a^* δ in ppm, *J* in Hz, TMS as the internal standard; *^b^* HMBC correlations, optimized for 6 Hz, were started from the proton to the indicated carbon; *^c^* signal partially obscured.

The relative configurations of the chiral centers of C-5′ to C-8′ in **2** were deduced from the coupling constants observed in the ^1^H NMR spectrum, as well as from the correlations detected in the NOESY spectrum (Figure 5b). The apparent coupling constant for 7′-H and 8′-H (*J* = 6.6 Hz) suggested a *trans-axial-pesudoaxial* relation of these protons [21]. Without commonly detected 5′-H/7′-H correlation in those of 5′-epimers [22], 7′-H further correlated with 5′-OH in the NOESY experiment, which suggested a *trans* relation between 5′-H and 7′-H. The methyl group at C-6′ was placed in an equatorial position by detected correlations of 6′-Me/7′-H and 6′-OH/8′-H. Hence, the relative configurations of **2** were 5′R*, 6′S*, 7′R*, 8′S*. The specific rotation value (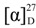 +75° (*c* 0.02, EtOH), 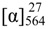 +208° (*c* 0.02, EtOH)) suggested that **2** was an atropisomer of alterporriol C [23]. Herein, we report the configuration of the chiral axis in the molecule by theoretical calculation.

**Figure 6:**
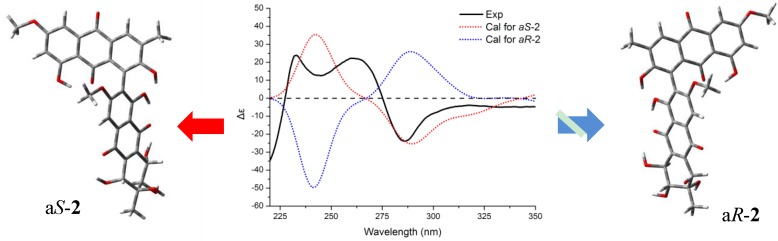
Experimental and calculated ECD spectra of **2** *.

The orientation of the biaryl axis has a drastic influence on the ECD spectrum, while central chirality has a marginal effect [17]. The application of the exciton chirality method to biphenanthryl derivatives had proven not to be straightforward, owing to the complex electronic structure of the chromophore [24]. Thus, ECD spectra were calculated for both a*R* (also defined as *P* helicity) and a*S* (also defined as *M* helicity) stereostructures and compared with the experimental spectrum measured in methanol for the assignment of the axial configuration of **2**. The complicated profile of the ^1^B_b_ transition region was observed; together with a couplet-like feature appearing as a strong negative couplet with a crossover at 275 nm. The conformational analysis and TDDFT methodology for ECD calculation of **2** were carried out as mentioned before for **1**. As a result, two low-energy conformers with energies that differed by less than 1 kcal/mol were obtained, each with a Boltzmann population above 30% at 300 K, which amounted to 90% overall population for both a*S* and a*R* configurations of **2**. The various minima, which differed in the orientation of the methoxy and hydroxy groups, had similar aryl-aryl torsions. For low-energy conformers of a*S*-atropisomer, the C2–C1–C2′–C1′ dihedral angle was −71.2° and −70.8°, respectively. For low-energy conformers of a*R*-atropisomer, the C2–C1–C2′–C1′ dihedral angles were 71.6° and 71.3°. The theoretical ECD spectrum for a*S*-**2** reproduced the experimental spectrum well in the decisive region of 225–325 nm [22] with negative CD absorption around 280 nm and positive CD absorption around 250 nm (Figure 6). Thus, the axial configuration of Compound **2** was identified as a*S*. It is worth mentioning that the shoulder peak around 230 nm was not reproduced well by B3LYP (this experiment), CAM-B3LYP/SVP and ZINDO/S-CI [22]; a more accurate method (e.g., “gold standard” CCSD(T)) could be expected to give a more sufficient result.

* Black solid line: ECD spectrum of **2** in methanol solution; red dotted line: calculated ECD spectrum for a*S*-**2** with the B3LYP/6-31G++(2d,p) method on the DFT-optimized structures as Boltzmann-weighted averages for two low-energy conformers; blue dotted line: calculated ECD spectrum for a*R*-**2** with the B3LYP/6-31G++(2d,p) method on the DFT-optimized structures as Boltzmann-weighted averages for two low-energy conformers. Spectra were generated as the sum of Gaussian bands with a 0.16 eV width and scaled.

The structures of known compounds hydroxybostrycin (**3**), halorosellinia A (**4**), tetrahydrobostrycin (**5**), 9α-hydroxydihydrodesoxybostrycin (**6**), austrocortinin (**7**) and 6-methylquinizarin (**8**) were identified by comparison of their spectroscopic data with those in the literature [11,12,23,25,26].

All compounds were investigated for their inhibitory activities against MptpB with sodium orthovanadate as the positive control (Table 3). The results showed that **2** was a potential inhibitor of MptpB with an IC_50_ value of 8.70 μM, which revealed that (+)-a*S*-alterporriol C (**2**) could be a potential antituberculosis drug and/or lead compound for constructing an antituberculosis compound library.

**Table 3:** *Mycobacterium* *tuberculosis* protein tyrosine phosphatase B (MptpB) assay for isolated Compounds **1**–**8**.

Compound	IC_50_ (μM)
**1**	64.70
**2**	8.70
**3–8**	>100.00
**Control**	0.05

## 3. Experimental Section

### 3.1. General

Optical rotations were determined with a RUDOLPH Autopol III polarimeter (Rudolph Research Analytical, Hackettstown, NJ, USA) at 27 °C. UV data were measured on a PERSEE TU-1900 spectrophotometer (Purkinje General Instrument Co., Ltd., Beijing, China). ECD data were recorded with a JASCO J–810 spectropolarimeter (JASCO, Inc., Easton, MD, USA). IR spectra were measured on a Nicolet Nexus 670 spectrophotometer (Thermo Fisher Scientific, Inc., Hudson, NH, USA), in KBr discs. ^1^H and ^13^C NMR data were recorded on a Bruker AVANCE 400 spectrometer (Bruker BioSpin Corporation, Billerica, MA, USA), respectively (TMS as the internal standard). ESIMS spectra were obtained from a Micro mass Q-TOF spectrometer (Waters Corporation, Milford, MA, USA). HRESIMS spectra were measured on a Thermo Scientific LTQ Orbitrap Elite high-resolution mass spectrometer (Thermo Fisher Scientific, Inc., Hudson, NH, USA). Silica gel (Qing Dao Hai Yang Chemical Group Co., Qingdao, China; 200–300 mesh), octadecylsilyl silica gel (Unicorn, Palarivattom, Kerala, India; 45 μm) and Sephadex LH-20 (GE Healthcare, Buckinghamshire, UK) were used for column chromatography. Precoated silica gel plates (Qing Dao Huang Hai Chemical Group Co., Qingdao, China; G60, F-254) were used for thin layer chromatography.

### 3.2. Strain Isolation, Taxonomic Classification and Endophyte Fermentation

The fungal strain SK11 was isolated from the root of *Excoecaria agallocha* from Shankou, Guangxi Province, China. It was identified as *Alternaria* sp. according to a molecular biological protocol by DNA amplification and sequencing of the ITS region (deposited in GenBank, accession No. EU807936). Voucher specimens are stored in the School of Chemistry and Chemical Engineering, Sun Yat-Sen University, Guangzhou, China, with the access code, SK11. The fungal strain was cultivated in potato glucose liquid medium (15 g glucose and 3 g sea salt in 1 L potato infusion) in 1 L Erlenmeyer flasks, each containing 300 mL of culture broth, at 26 °C without shaking for 4 weeks.

### 3.3. Extraction and Separation of Metabolites

The culture (100 L) was filtered to separate the culture broth from the mycelia. The culture broth was extracted three times with an equal volume of EtOAc. The combined EtOAc layers were evaporated to dryness under reduced pressure to give an EtOAc extract (20.5 g), which was subjected to silica gel column chromatography (CC) (petroleum ether, EtOAc v/v, gradient) to generate six fractions (Fr. 1–6). Fr. 5 was isolated by CC on silica gel eluted with methylene chloride-MeOH, then subjected to Sephadex LH-20 CC eluting with MeOH and further purified by using an ODS semi-preparative column eluted with 45%–65% MeOH–H_2_O to obtain Compounds **1** (2.1 mg) and **2** (4.5 mg).

Compound **1:** Red powder (MeOH); 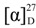 +40° (*c* 0.02, EtOH); UV (MeOH) λ_max_ 362.2, 288.8, 248.6, 220.4 nm; CD (MeOH) Δε_355_ (−3.3), Δε_2__93_ (+8.8), Δε_2__54_ (−5.1), Δε_2__35_ (+1.6), Δε_2__10_ (−18.6) cm^2^ mol^−1^; IR (KBr) ν_max_ 3432, 2916, 2857, 1653, 1437, 1383, 1077, 480 cm^−^^1^; For ^1^H, ^13^C and 2D NMR spectroscopic data, see Table 1; HRESIMS *m*/*z* = 611.1793 (calcd. for C_31_H_31_O_13_, 611.1759).

Compound **2:** Orange powder (MeOH); 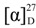 +75° (*c* 0.02, EtOH), 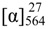 +208° (*c* 0.02, EtOH); UV (MeOH) λ_max_ 314.6, 280.4, 256.8, 226.4 nm; CD (MeOH) Δε_285_ (−23.9), Δε_259_ (+22.2), Δε_245_ (+12.2), Δε_233_ (+25.1), Δε_218_ (−39.5) cm^2^ mol^−1^; IR (KBr) ν_max_ 3406, 2919, 2519, 1604, 1429, 1384, 1284, 1161, 1070, 878, 711, 613, 546, 483 cm^−1^; for ^1^H, ^13^C and 2D NMR spectroscopic data, see Table 2; HRESIMS *m*/*z* = 617.1335 (calcd. for C_32_H_25_O_13_, 617.1290).

### 3.4. Calculation of ECD Spectra

Molecular mechanics calculations were run with Spartan ′10 (Wavefunction, Inc., Irvine, CA, USA) with standard parameters and convergence criteria. DFT and TDDFT calculations were run with Gaussian 03 (Gaussian, Wallingford, CT, USA) with default grids and convergence criteria. TDDFT calculations were carried out by using the b3lyp/6-311++g(2d,p) or b3lyp/6-31++g(2d,p) method and included 10 single excited states in each case. The IEF-PCM solvent model for methanol was included in all cases. ECD spectra were generated using the programs, SpecDis 1.6 (University of Würzburg, Würzburg, Germany) and OriginPro 8.5 (OriginLab, Ltd., Northampton, MA, USA), by applying Gaussian band shapes with 0.16 eV exponential half-width from dipole-length rotational strengths. All calculations were performed with the High-Performance Grid Computing Platform of Sun Yat-Sen University.

### 3.5. Materials and Methods for mPTPB Assay

#### 3.5.1. Cloning, Expression and Purification of MptpB

The full-length PTPB gene was amplified from genomic DNA of the *Mtb* H37Rv strain (School of Life Sciences, Sun Yat-Sen University: Guangzhou, China). PCR products were cloned in frame with an N-terminal His_6_ tag into the pET28a (+) vector (Novagen, Merck KGaA, Darmstadt, Germany). For protein expression, plasmids were transformed into *Escherichia coli* BL21(DH3) cells (Invitrogen, Thermo Fisher Scientific, Inc., Hudson, NH, USA) and grown in LB medium containing 50 μg/mL kanamycin at 37 °C till the OD_600_ of the solution was about 0.6. After the addition of 0.1 mM IPTG, the culture was grown for another 16 h at 20 °C. The cells were harvested by centrifugation at 5000 rpm for 5 min at 4 °C. The bacterial cell pellets were resuspended in the buffer containing 20 mM Tris, pH 7.9, 500 mM NaCl and 5 mM imidazole and were lysed by sonication on ice. Cellular debris was removed by centrifugation at 10,000 rpm for 30 min at 4 °C. The protein was purified from the supernatant using glutathione-Sepharose 4B (GE Healthcare, Buckinghamshire, UK), according to the manufacturer’s instructions. Protein concentration was determined using the Bradford dye binding assay (Bio-Rad Laboratories, Inc, Hercules, CA, USA), according to the manufacturer’s recommendations, with bovine serum albumin as the standard. The purified MptpB was stored in 30% glycerol at −20 °C.

#### 3.5.2. MptpB Inhibition Assay

The inhibition assays were performed using the RediPlate 96 EnzChek Tyrosine Phosphatase Assay kit (Invitrogen, Thermo Fisher Scientific, Inc., Hudson, NH, USA) by monitoring the hydrolysis of the fluorogenic phosphatase substrate, 6,8-difluoro-methylumbelliferyl phosphate (DiFMUP), according to the manufacturer’s instruction. The IC_50_ value was determined at five different substrate concentrations by non-linear regression fitting of the inhibitor concentration *vs.* inhibition rate. All measurements were done in triplicate from two independent experiments. The reported IC_50_ was the average value of two independent experiments.

## 4. Conclusions

*Alternaria* sp. (SK11), an endophytic fungus from the South China Sea, was proven as a prolific producer of bioactive metabolites. Eight compounds were isolated from this fungal strain, including two alterporriol-type dimers, the relative and absolute configurations of which were established by using spectral and theoretical methods. The novel compound, alterporriol S (**1**), was the first member of alterporriols with a unique C-10−C-2′ linkage [20,22,23,26,27,28,29,30,31,32,33]. Anthranoid dimers consisting of such a coupling type could only be found in a small population of terrestrial plants [14,15,34,35,36,37,38,39,40,41,42,43] and marine animal [44]; this was the first report of a fungus origin. In the bioassay, (+)-a*S*-alterporriol C (**2**) showed strong inhibitory activity against MptpB, which indicated that it could represent a new type of lead compounds for the development of new anti-tuberculosis drugs. The *in vitro* inhibition, crystallization, *in vivo* racemization rates and ADMET (absorption, distribution, metabolism, excretion and toxicity) properties of atropisomers are frequently attributed to their axial configuration [45]. We determined the configuration of chiral axis in **2** as a*S* for the first time, and the ultimate assignment of the chiral centers may require the use of an alternative technique, like vibrational circular dichroism [46].

## Supplementary Files

Supplementary File 1 PDF-Document (PDF, 1589 KB)

## Abbreviation

B3LYP: Becke-3-Parameter-Lee-Yang-Parr
CAM–B3LYP: Coulomb Attenuating Method based on B3LYP
CCSD(T): Coupled-Cluster Singles and Doubles (Triple)
Fr.: Fraction
IEF-PCM: Integral-Equation-Formalism Polarizable Continuum Model
ITS: Internal Transcribed Spacer
SVP: Split Valence plus Polarization
TMS: Tetramethylsilane
ZINDO/S-CI: Zerner’s Intermediate Neglect of Differential Overlap-Spectroscopic-Configuration Interaction
