# Supplementary material for: Alterporriol-Type Dimers from the Mangrove Endophytic Fungus, Alternaria sp. (SK11), and Their MptpB Inhibitions
Source: Mar Drugs. 2014 May 16;12(5):2953–69. doi: 10.3390/md12052953 (PMC4052326; doi:10.3390/md12052953)
Supplement: Supplementary File 1 — PDF-Document (PDF, 1589 KB) [file marinedrugs-12-02953-s001.pdf]

Supplementary Information

Figure S1. ^{1}H NMR spectrum of **1** (400 MHz, DMSO-d_6).
Figure S2. ^{13}C NMR spectrum of **1** (100 MHz, DMSO-d_6).
Figure S3. DEPT (135°) spectrum of **1** (400 MHz, DMSO-d_6).
Figure S4. DEPT (90°) spectrum of **1** (400 MHz, DMSO-d_6).
Figure S5. ^{1}H-^{1}H COSY spectrum of **1** (400 MHz, DMSO-d_6).
Figure S6. HSQC spectrum of **1** (400/100 MHz, DMSO-d_6).
Figure S7. HMBC spectrum of **1** (400/100 MHz, DMSO-d_6).
Figure S8. NOESY spectrum of **1** (400 MHz, DMSO-d_6).
Figure S9. HRESIMS spectrum of **1**.
Figure S10. DFT-optimized low-energy conformations for 6*S*, 7*R*, 8*S*, 8a*S*, 10*R*, 10a*R*, 6′*R*, 7′*S*-**1**.
Table S1. Ground to excited state transition dipole moments calculated with TDDFT for global minima **1-1**.
Table S2. Ground to excited state transition dipole moments calculated with TDDFT for global minima of 6*S*, 7*R*, 8*S*, 8a*S*, 10*R*, 10a*R*, 6′*S*, 7′*R*-**1**.
Table S3. TDDFT calculated rotatory strengths and oscillator strengths for global minima **1-1**.
Chart S1. Stimulated CD spectra for low-energy conformers of 6*S*, 7*R*, 8*S*, 8a*S*, 10*R*, 10a*R*, 6′*R*, 7′*S*-**1**.
Chart S2. Stimulated CD spectra for low-energy conformers of 6*S*, 7*R*, 8*S*, 8a*S*, 10*R*, 10a*R*, 6′*S*, 7′*R*-**1**.
Chart S3. The most relevant occupied and unoccupied molecular orbitals of 1-1 for $S_0 \rightarrow S_3$ and $S_0 \rightarrow S_4$ transitions.
Table S4. Identification of the most relevant excitations of **1-1** as calculated by the TDB3LYP/6-311++G(2d,p) method.
Figure S11. ^{1}H NMR spectrum of **2** (400 MHz, DMSO-d_6).
Figure S12. ^{13}C NMR spectrum of **2** (100 MHz, DMSO-d_6).
Figure S13. DEPT (135°) spectrum of **2** (400 MHz, DMSO-d_6).
Figure S14. ^{1}H-^{1}H COSY spectrum of **2** (400 MHz, DMSO-d_6).
Figure S15. HSQC spectrum of **2** (400/100 MHz, DMSO-d_6).
Figure S16. HMBC spectrum of **2**(400/100 MHz, DMSO-d_6).
Figure S17. NOESY spectrum of **2** (400 MHz, DMSO-d_6).
Figure S18. HRESIMS spectrum of **2**.
Figure S19. DFT-optimized low-energy conformations for a*S*-**2**.
Table S5. TDDFT calculated rotatory strengths and oscillator strengths for global minima **2-1**.

Figure S1. ^{1}H NMR spectrum of **1** (400 MHz, DMSO-d_6).

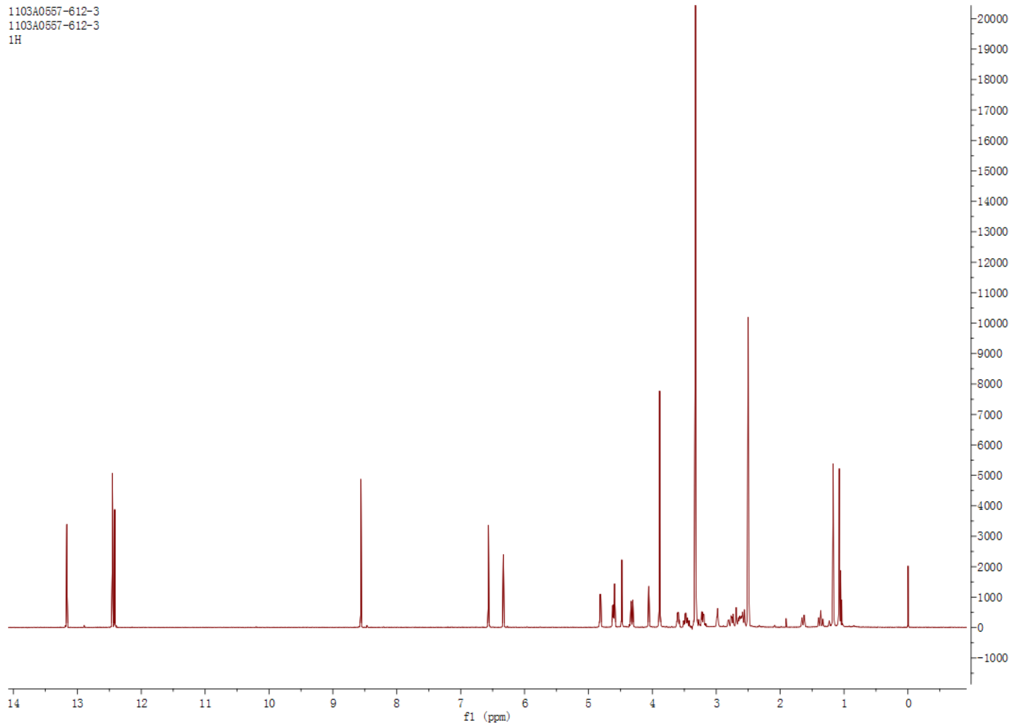

Figure S2. ^{13}C NMR spectrum of **1** (100 MHz, DMSO-d_6).

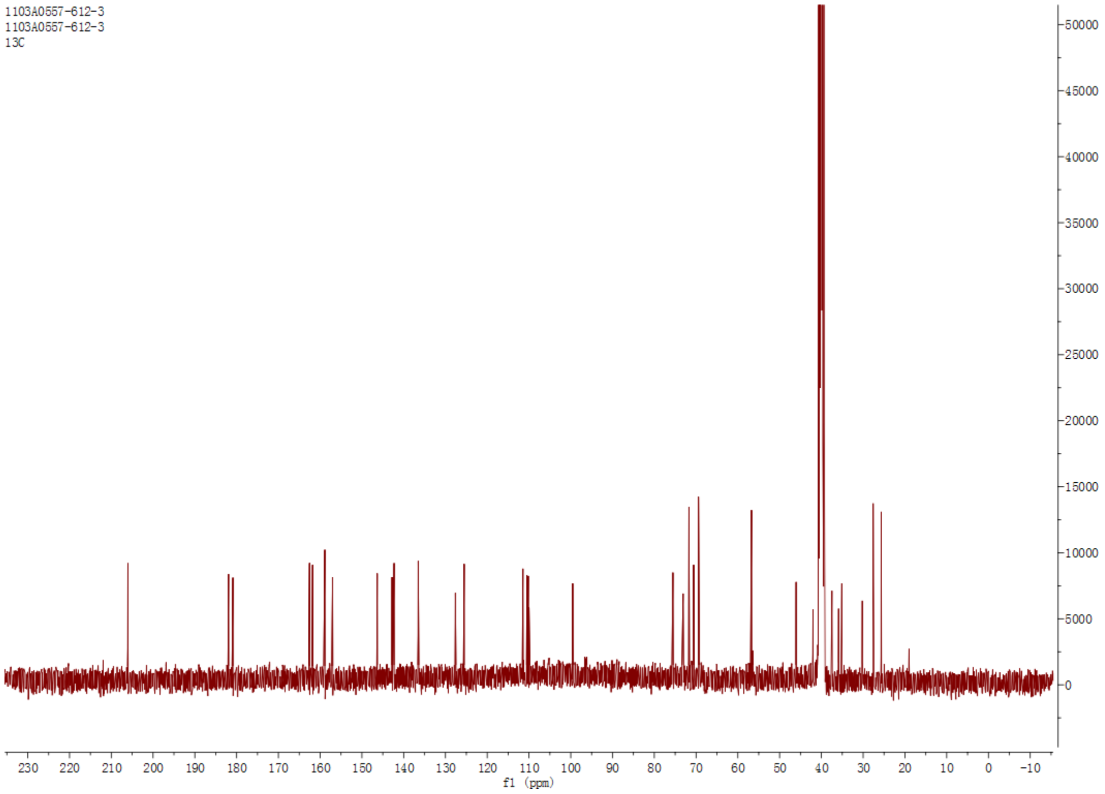

Figure S3. DEPT (135°) spectrum of **1** (400 MHz, DMSO-d_6).

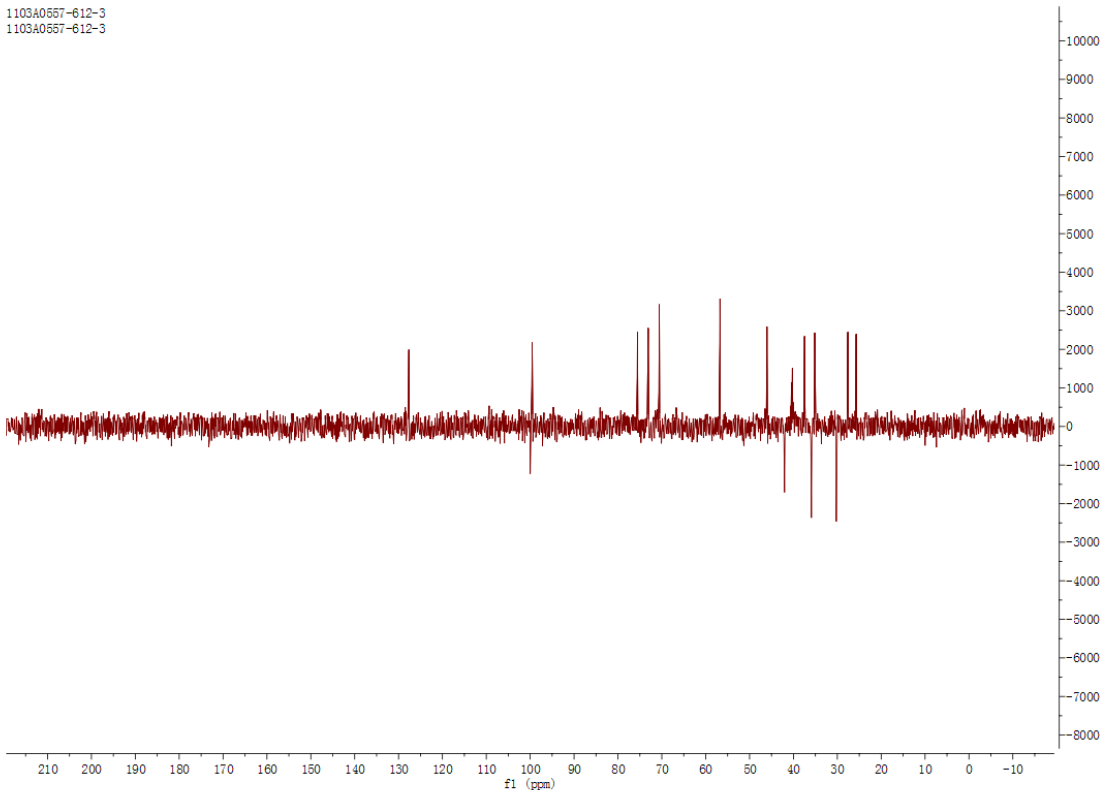

Figure S4. DEPT (90°) spectrum of **1** (400 MHz, DMSO-d_6).

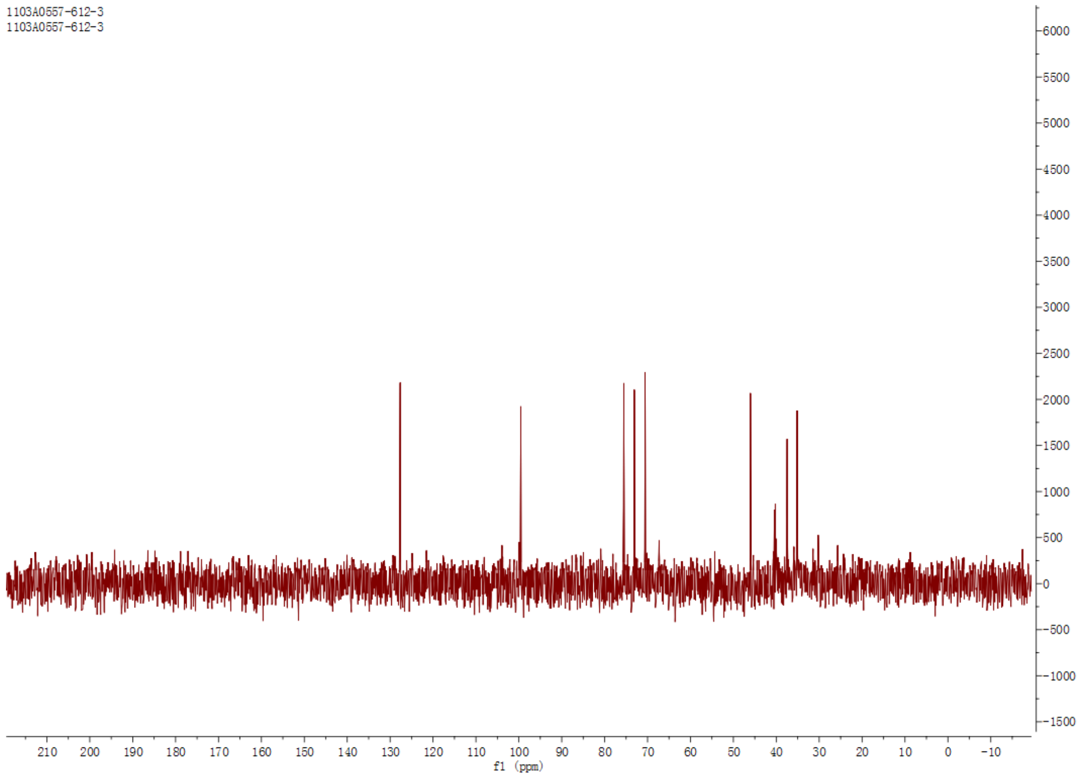

Figure S5. ^{1}H–^{1}H COSY spectrum of **1** (400 MHz, DMSO-d_6).

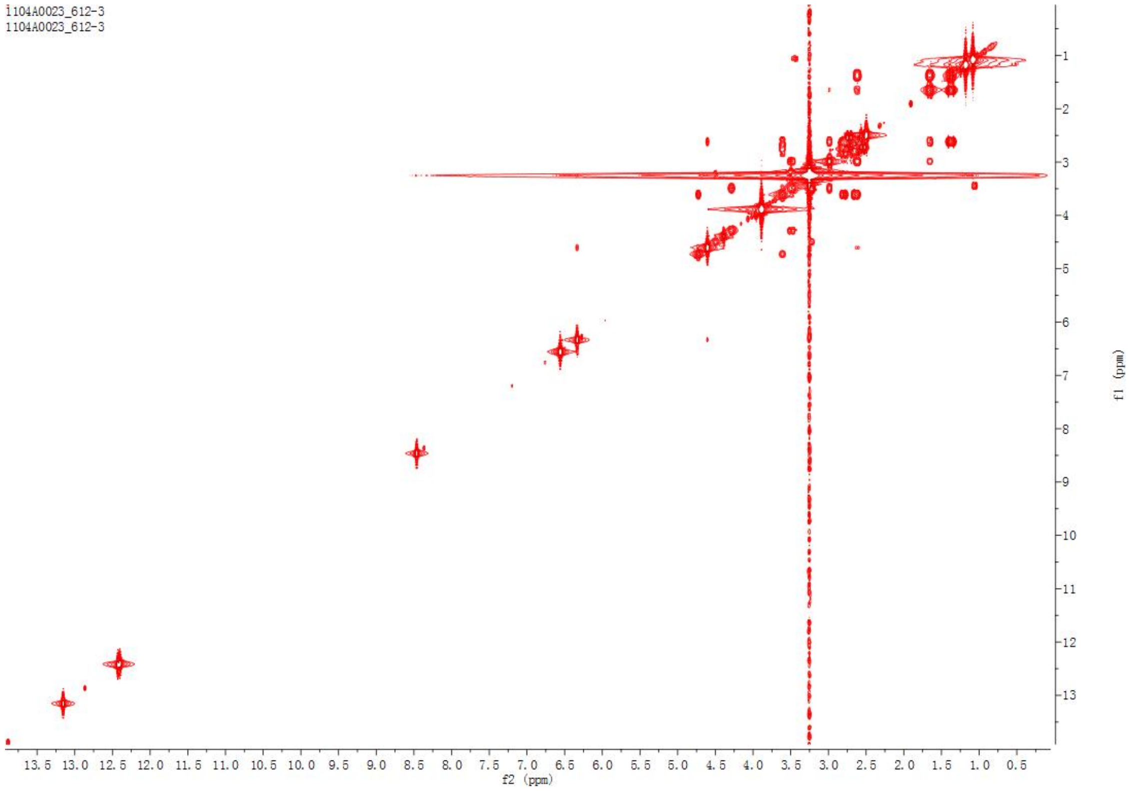

Figure S6. HSQC spectrum of **1** (400/100 MHz, DMSO-d_6).

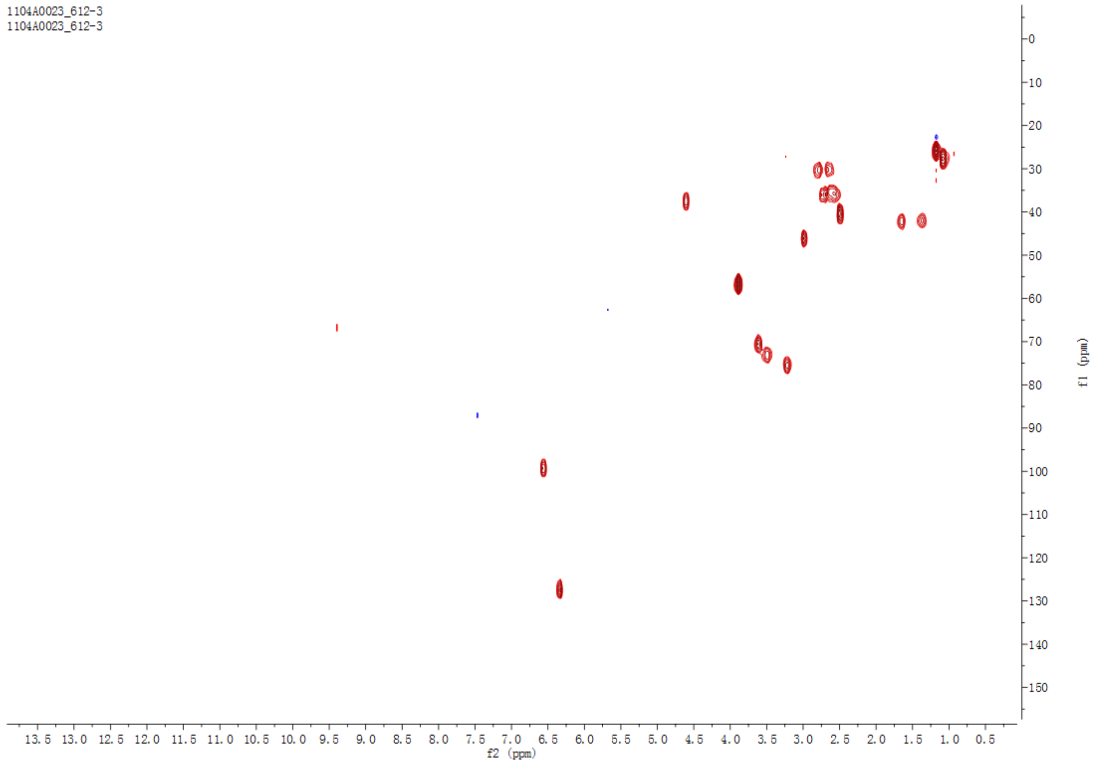

Figure S7. HMBC spectrum of **1** (400/100 MHz, DMSO-d_6).

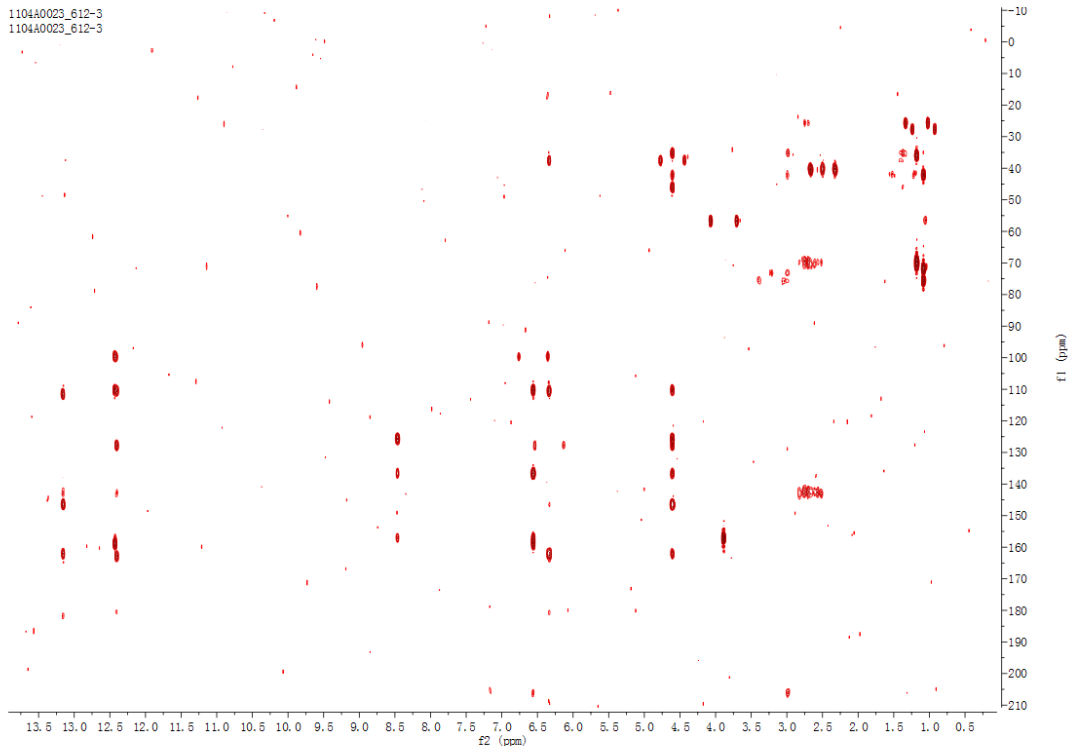

Figure S8. NOESY spectrum of **1** (400 MHz, DMSO-d_6).

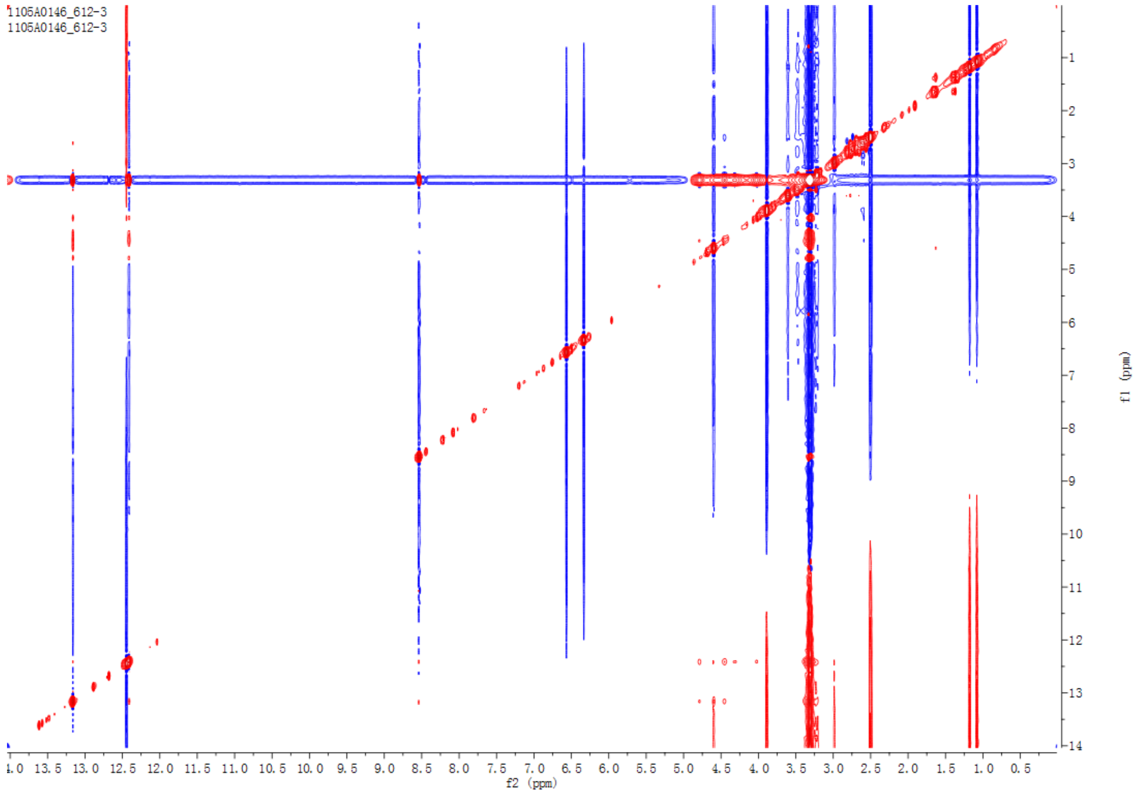

Figure S9. HRESIMS spectrum of **1.**

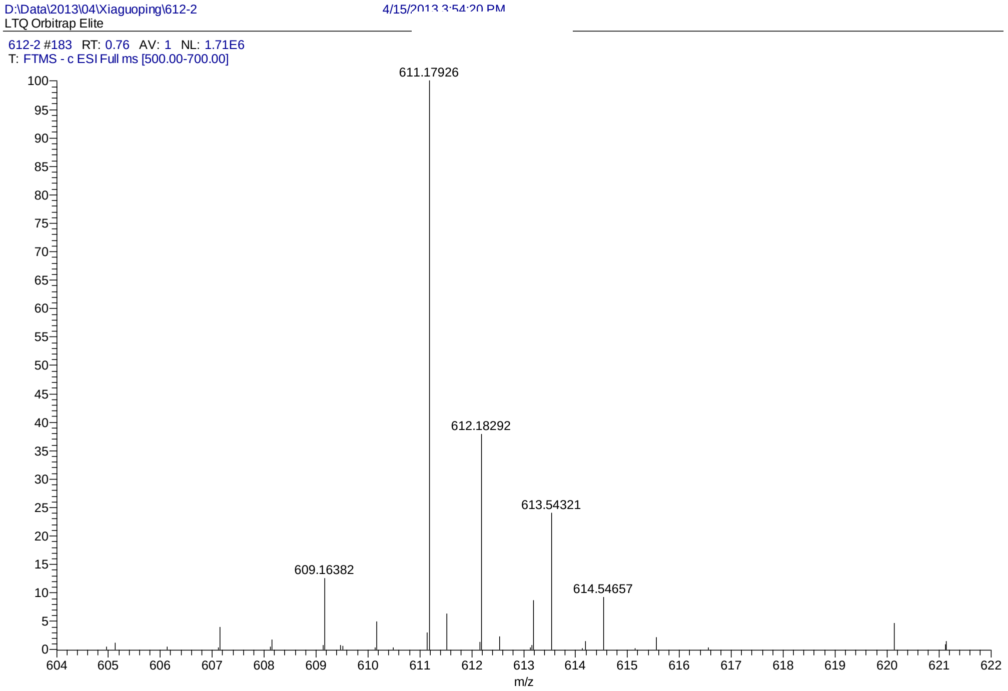

SPECTRUM-simulation:

m/z	Theoretical Mass	Delta (ppm)	RDB Equivalent	Composition
611.17926	611.17592	5.47	16.5	C31 H31 O13

Elements in use: ^{12}C (1~40), ^{16}O (1~20), ^{1}H (1~80).

Figure S10. DFT-optimized low-energy conformations (**1-1**–**1-4**) for 6*S*, 7*R*, 8*S*, 8a, 10*R*, 10a*R*, 6′*R*, 7′*S*-**1**.

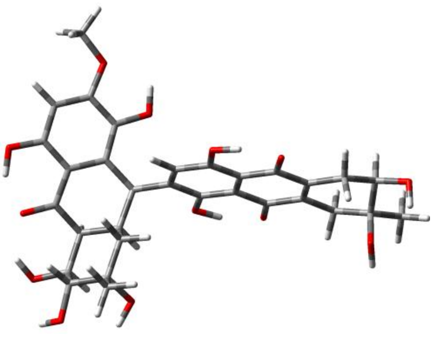

1-1 (ΔE = 0 Kcal/mol)

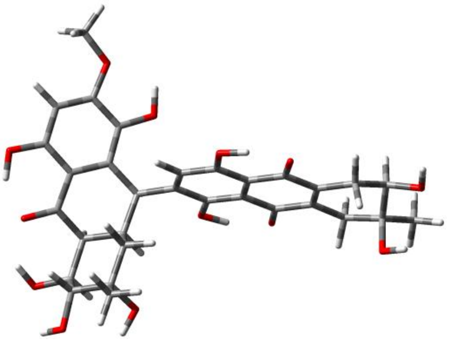

1-2 (ΔE = 0.06 Kcal/mol)

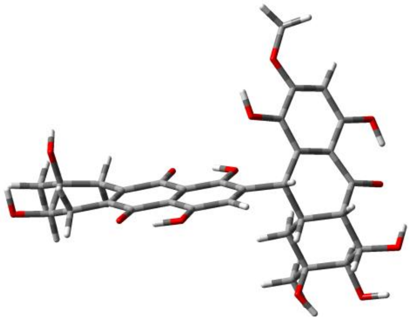

1-3 (ΔE = 1.16 Kcal/mol)

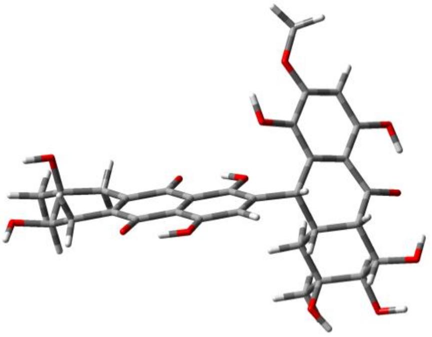

1-4 (ΔE = 1.18 Kcal/mol)

Table S1. Ground to excited state transition dipole moments calculated with TDDFT for global minima **1-1**.

State	Electric Dipole Moments (Au)				Velocity Dipole Moments (Au)				Magnetic Dipole Moments (Au)		
	X	Y	Z	Dip. S.	X	Y	Z	Dip. S.	X	Y	Z
1	0.3536	−0.1076	0.0289	0.1375	−0.0271	0.0083	−0.0021	0.0008	0.0106	0.0027	−0.0079
2	−1.9718	0.1638	0.0974	3.9244	0.1801	−0.0148	−0.0087	0.0327	−0.0032	0.1096	−0.2142
3	−0.0215	0.0019	−0.0143	0.0007	0.0022	−0.0005	0.0018	0.0000	−0.0772	−0.5129	−0.2929
4	−0.0735	−0.0018	−0.0113	0.0055	0.0085	0.0001	0.0015	0.0001	0.0305	0.7935	−0.5700
5	−0.0831	−0.0563	−0.1018	0.0204	0.0095	0.0065	0.0118	0.0003	−0.0672	−0.5616	0.1390
6	0.0439	−0.0488	−0.0273	0.0051	−0.0055	0.0061	0.0035	0.0001	0.0862	0.0296	−0.0247
7	0.4275	−1.2836	−0.0752	1.8362	−0.0544	0.1606	0.0095	0.0288	0.2697	0.2460	−0.7086
8	0.0384	0.0721	0.0306	0.0076	−0.0051	−0.0094	−0.0038	0.0001	0.0318	0.0617	−0.0443
9	−0.0446	0.0668	0.0779	0.0125	0.0059	−0.0087	−0.0102	0.0002	0.0912	0.1097	0.0067
10	−0.1162	−0.2352	−0.1151	0.0821	0.0161	0.0323	0.0157	0.0015	0.0551	−0.0988	−0.0864

Table S2. Ground to excited state transition dipole moments calculated with TDDFT for global minima of 6*S*, 7*R*, 8*S*, 8a*S*, 10*R*, 10a*R*, 6′*S*, 7′*R*-**1**.

State	Electric Dipole Moments (Au)				Velocity Dipole Moments (Au)				Magnetic Dipole Moments (Au)		
	X	Y	Z	Dip. S.	X	Y	Z	Dip. S.	X	Y	Z
1	0.3330	−0.1131	0.0319	0.1247	−0.0255	0.0087	−0.0023	0.0007	0.0121	0.0044	−0.0176
2	−1.9705	0.2100	0.0811	3.9334	0.1800	−0.0191	−0.0072	0.0328	0.0045	0.1171	−0.1914
3	0.0165	0.0206	0.0415	0.0024	−0.0018	−0.0027	−0.0045	0.0000	−0.0517	−0.0805	−0.5116
4	0.0625	−0.0026	0.0076	0.0040	−0.0073	0.0003	−0.0010	0.0001	−0.0735	−0.9074	0.4120
5	−0.0749	−0.0501	−0.0883	0.0159	0.0086	0.0057	0.0102	0.0002	−0.0906	−0.6011	0.0906
6	0.0642	−0.1215	0.0225	0.0194	−0.0081	0.0151	−0.0028	0.0003	0.0992	0.0394	−0.0794
7	0.3908	−1.2905	−0.0919	1.8266	−0.0498	0.1614	0.0116	0.0287	0.2691	0.2461	−0.7048
8	0.0421	0.0690	0.0292	0.0074	−0.0055	−0.0090	−0.0037	0.0001	0.0332	0.0572	−0.0367
9	0.0481	−0.0419	−0.1054	0.0152	−0.0063	0.0056	0.0138	0.0003	0.1099	−0.0938	0.1054
10	−0.1428	−0.2347	−0.1518	0.0985	0.0197	0.0322	0.0208	0.0019	0.0469	−0.1475	−0.0791

Table S3. TDDFT calculated rotatory strengths and oscillator strengths for global minima **1-1**.

1/2[<0|r|b>*<b|rxdel|0> + (<0|rxdel|b>*<b|r|0>)*]

Rotatory Strengths (R) in cgs (10**-40 erg-esu-cm/Gauss)

State	XX	YY	ZZ	R (length)	R (au)
1	−2.6584	0.2039	0.1615	−0.7644	−0.0016
2	−4.4039	−12.6917	14.7576	−0.7794	−0.0017
3	−1.1760	0.6913	−2.9635	−1.1494	−0.0024
4	1.5885	1.0127	−4.5694	−0.6561	−0.0014
5	−3.9481	−22.3673	10.0022	−5.4377	−0.0115
6	−2.6719	1.0203	−0.4771	−0.7096	−0.0015
7	−81.5216	223.3304	−37.6938	34.7050	0.0736
8	−0.8622	−3.1446	0.9582	−1.0162	−0.0022
9	2.8772	−5.1838	−0.3670	−0.8912	−0.0019
10	4.5259	−16.4283	−7.0309	−6.3111	−0.0134

Table S3. *Cont.*

	1/2[<0|del|b>*<b|r|0> + (<0|r|b>*<b|del|0>)*] (Au)				
**State**	**X**	**Y**	**Z**	**Dipole Strength**	**Oscillator (Frdel)**
1	−0.0096	−0.0009	−0.0001	0.0105	0.0070
2	−0.3551	−0.0024	−0.0008	0.3584	0.2389
3	0.0000	0.0000	0.0000	0.0001	0.0001
4	−0.0006	0.0000	0.0000	0.0006	0.0004
5	−0.0008	−0.0004	−0.0012	0.0024	0.0016
6	−0.0002	−0.0003	−0.0001	0.0006	0.0004
7	−0.0232	−0.2061	−0.0007	0.2300	0.1534
8	−0.0002	−0.0007	−0.0001	0.0010	0.0007
9	−0.0003	−0.0006	−0.0008	0.0016	0.0011
10	−0.0019	−0.0076	−0.0018	0.0113	0.0075

Quantum mechanically, each electronic transition between two distinct electronic states generated different electric fields (***m***) and magnetic fields (***μ***). By calculating the electric ($\overrightarrow{\mathbf{m}_{fi}}\neq 0$) and magnetic transition dipole ($\overrightarrow{\boldsymbol{\mu}_{fi}}\neq 0$), which were allied to electron cloud redistribution taking place during the transition, the rotatory strengths (R_{fi}) of each state were generated (Equation (1)), which were defined to indicate the CE observed in the relevant regions (Equation (2)) [1,2].

$$R_{fi}=\mathrm{Im}(\boldsymbol{\mu}_{fi}\cdot\mathbf{m}_{fi})=\left|\overrightarrow{\mu_{fi}}\right|\cdot\left|\overrightarrow{m_{fi}}\right|\cdot\cos\left(\overrightarrow{\mu_{fi}},\overrightarrow{m_{fi}}\right) \quad (1)$$

$$R=\frac{2.303\cdot 3000\cdot hc}{32\pi^3 N_A}\int\frac{\Delta\varepsilon}{\lambda}d_\lambda \quad (2)$$

It is critical that an electric and a magnetic transition dipole defined by each electronic transition were not orthogonal (Table S1, Table S2). By defining the angle between the two dipoles ($\cos\left(\overrightarrow{\mu_{fi}},\overrightarrow{m_{fi}}\right)\neq 0$), *R* of diastereomers was of the opposite sign, indicating negative and positive CE in the relevant region, respectively (Table S3).

Chart S1. Stimulated CD spectra for low-energy conformers of 6*S*, 7*R*, 8*S*, 8a*S*, 10*R*, 10a*R*, 6′*R*, 7′*S*-**1**.

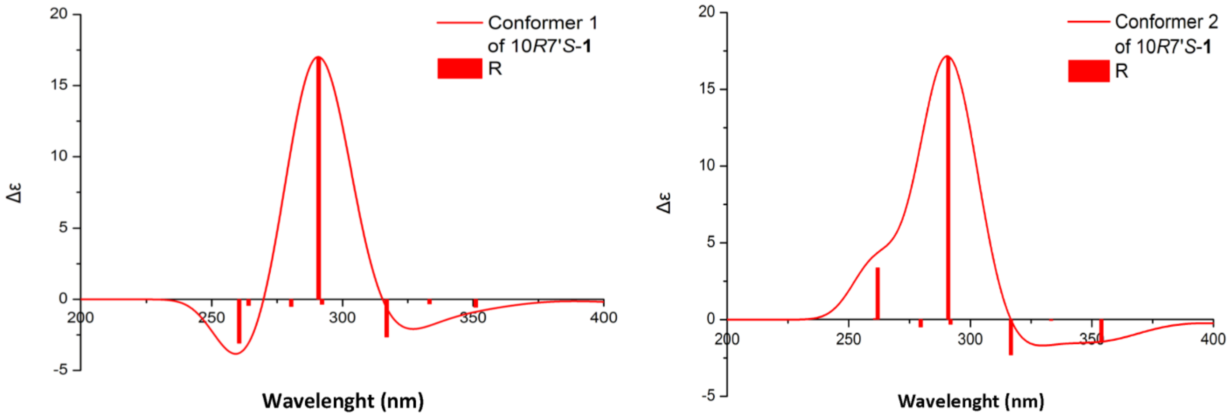

Chart S1. *Cont.*

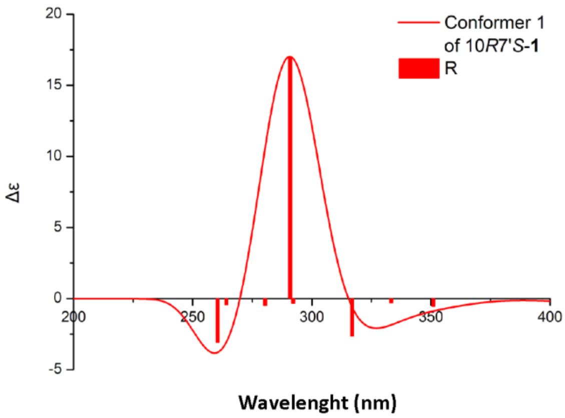

Chart S2. Stimulated CD spectra for low-energy conformers of 6*S*, 7*R*, 8*S*, 8a*S*, 10*R*, 10a*R*, 6′*S*, 7′*R*-**1**.

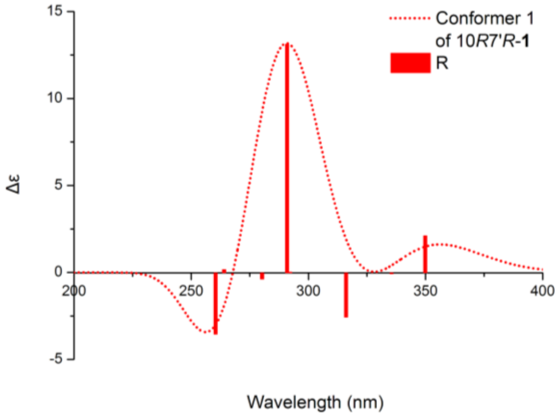

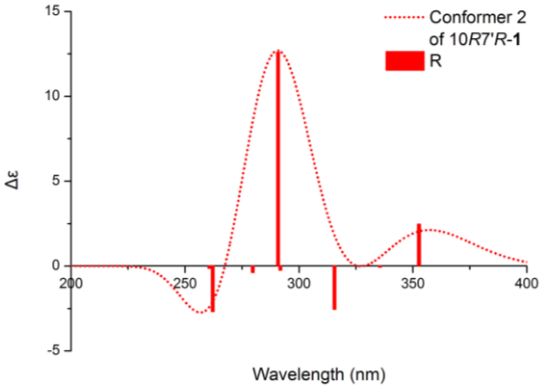

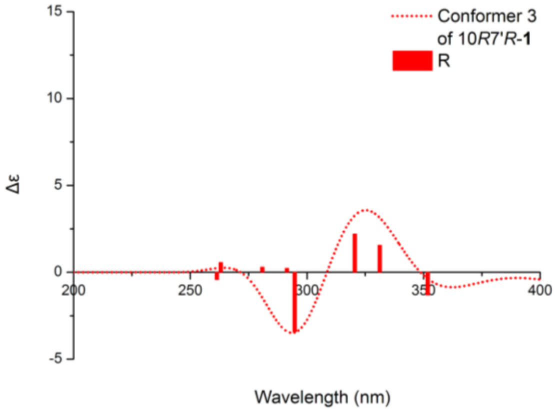

Chart S3. The most relevant occupied and unoccupied molecular orbitals (MOs) of **1-1** for $S_0 \rightarrow S_3$ and $S_0 \rightarrow S_4$ transitions.

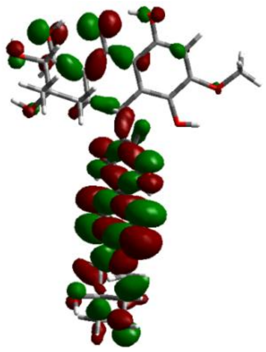

156 ($HOMO_{-5}$)

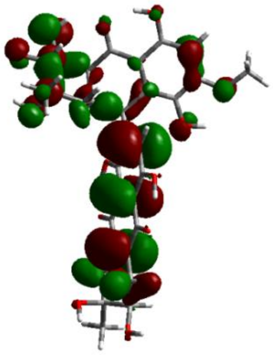

158 ($HOMO_{-3}$)

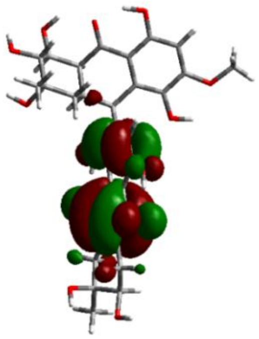

162 (LUMO)

Table S4. Identification of the most relevant excitations of **1-1** as calculated by the TDB3LYP/6-311++G(2d,p) method.

State	ΔE (eV)	*f*	$R \cdot 10^{-40}$ ($erg \cdot cm^3$)	Transition	Weight	Transition Dipole (au) [a]		
						X	Y	Z
				155→162	6.06%	0.035772	0.082408	0.144359
				156→162	69.53%	−0.123833	−0.163566	−0.360379
S_3	2.9312	0.0001	−1.1494	157→162	3.85%	−0.010089	−0.011743	−0.009224
				158→162	4.33%	0.005645	0.027352	0.029306
				159→162	12.23%	0.044599	0.040925	0.073040
						−0.047907	−0.024625	−0.122898
				155→162	8.54%	0.042459	0.097813	0.171345
				156→162	10.84%	−0.048897	−0.064587	−0.142301
S_4	3.0588	0.0004	−0.6561	157→162	2.44%	0.008035	0.009351	0.007346
				158→162	48.51%	−0.018903	−0.091597	−0.098140
				159→162	27.75%	−0.067198	−0.061662	−0.110050
						−0.084504	−0.110681	−0.171800

[a] Transition dipole moments for each pair of MOs were calculated with Multiwfn 3.3 [3].

For **1-1** (Figure S10), the most relevant excited states involved in the first CD absorption were $S_0 \rightarrow S_3$ and $S_0 \rightarrow S_4$. The nature of the excited states giving rise to the CE in 315–400 nm was analyzed. As shown above, the CE had mostly the character of n–π*- and π–π*-type transitions, which mainly participated in the southern moiety of the molecular. The most relevant occupied molecular orbital 156 ($HOMO_{–5}$) possessed complex shapes, representing actually a conjugation of n-orbitals of the oxygen atoms, the carbonyl and the hydroxyl, with the σ-type orbitals of the saturated ring. While in the case of the orbital 158 ($HOMO_{–3}$), π-type orbitals of the ring interacts with σ orbitals. The lowest unoccupied molecular orbital162 (LUMO) was a π* orbital of the aromatic ring combined with π* MOs of the carbonyl functions at C-9′ and C-10′. For more effective analysis, NTO (natural transition orbital) formalism [4] should be used.

Figure S11. ^{1}H NMR spectrum of **2** (400 MHz, DMSO-d_6).

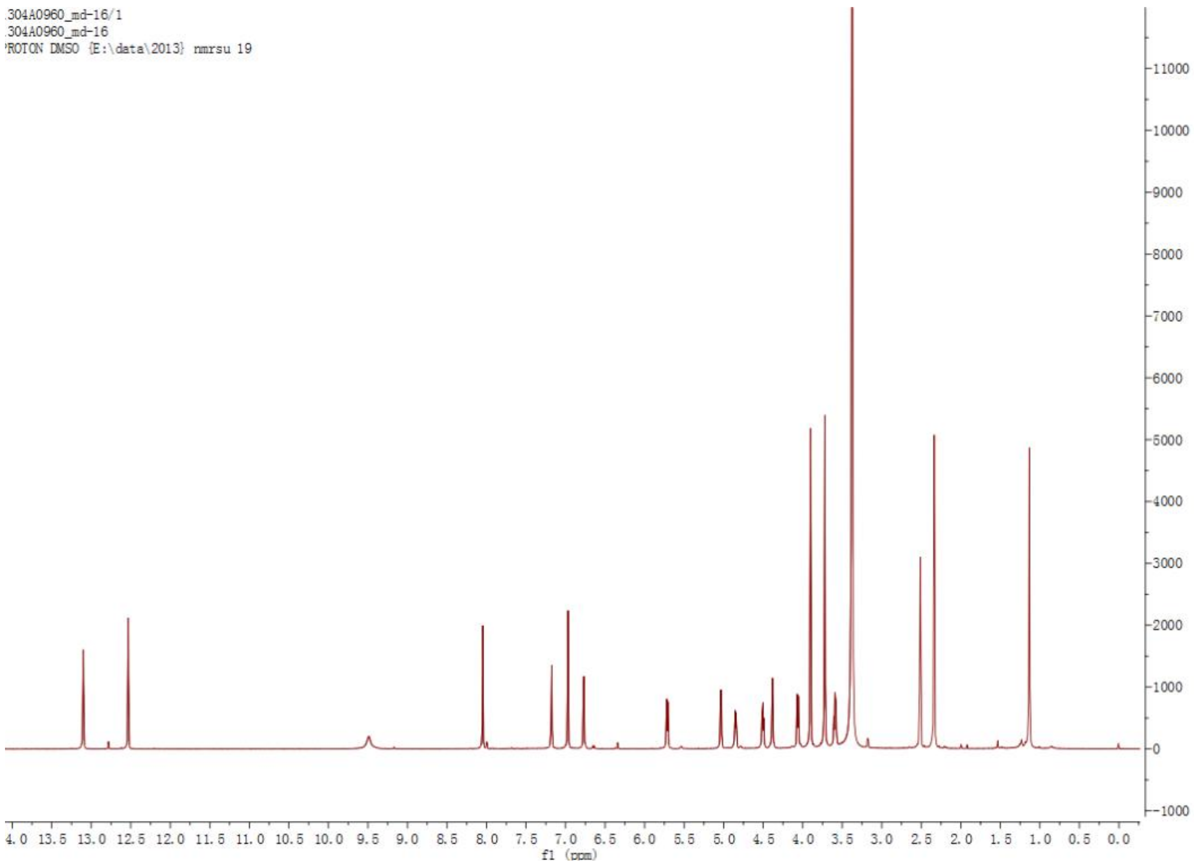

Figure S12. ^{13}C NMR spectrum of **2** (100 MHz, DMSO-d_6).

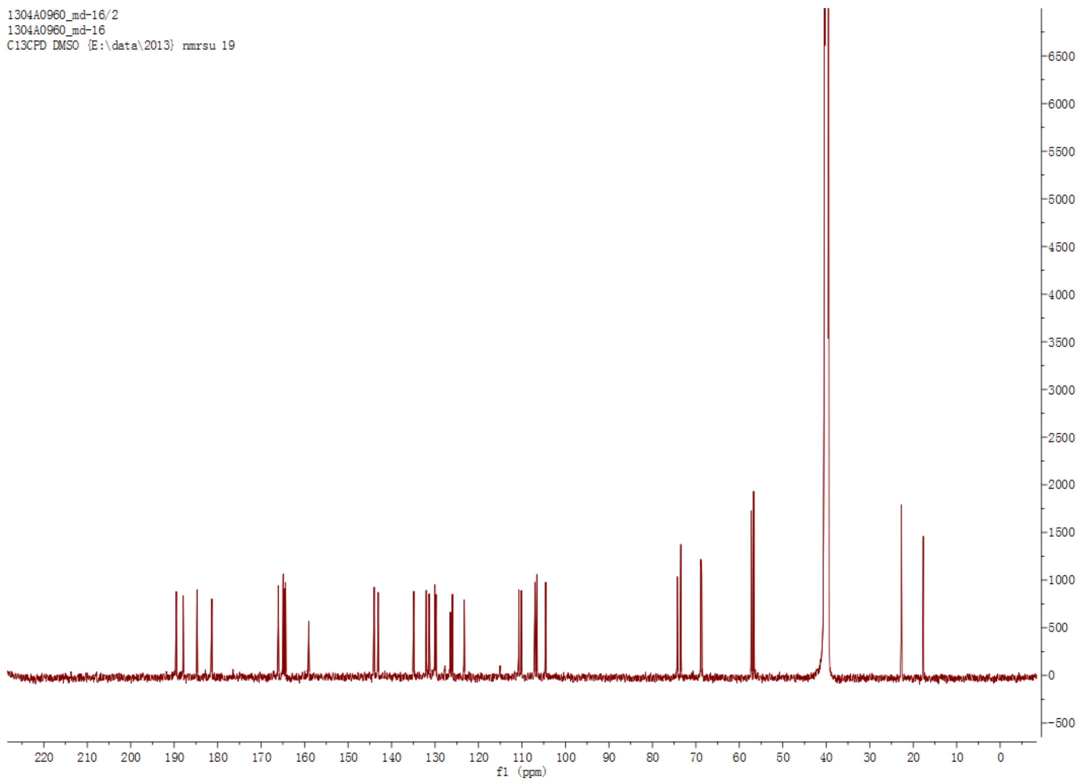

Figure S13. DEPT (135°) spectrum of **2** (400 MHz, DMSO-d_6).

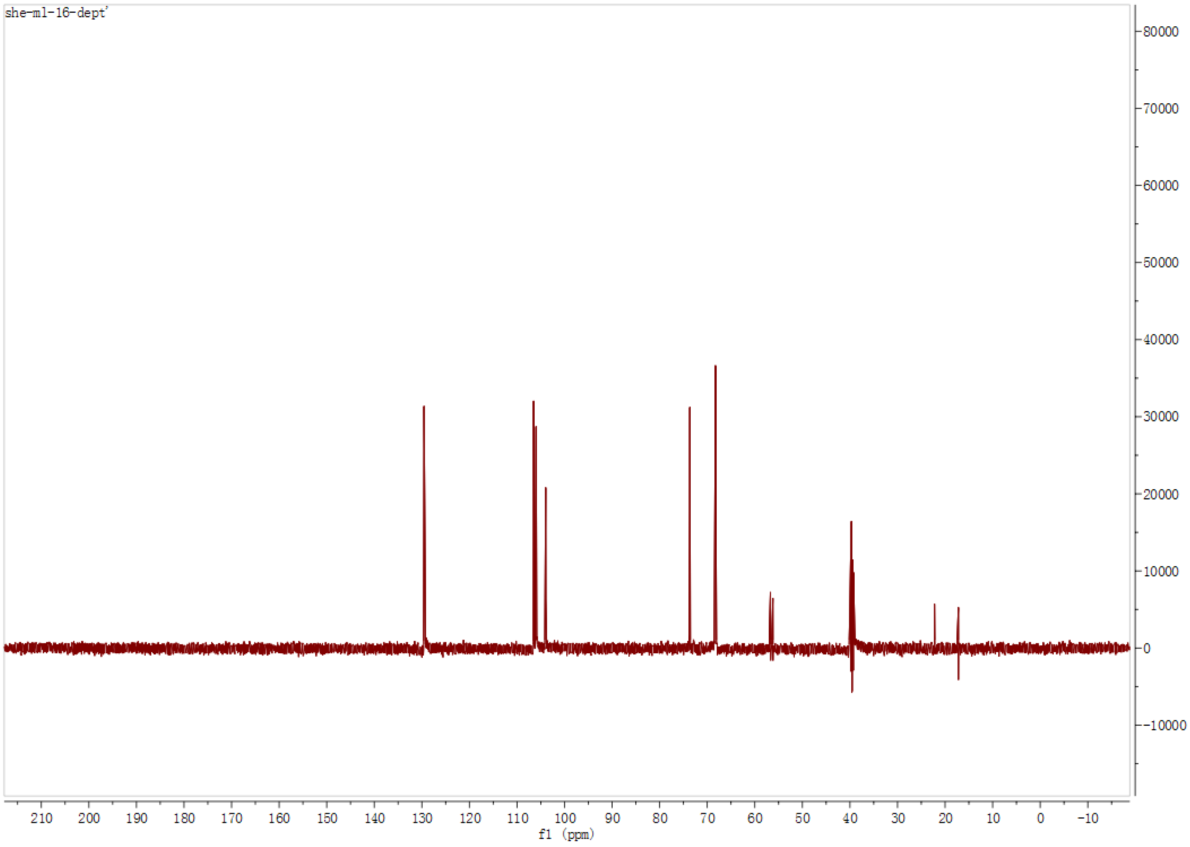

Figure S14. ^{1}H–^{1}H COSY spectrum of **2** (400 MHz, DMSO-d_6).

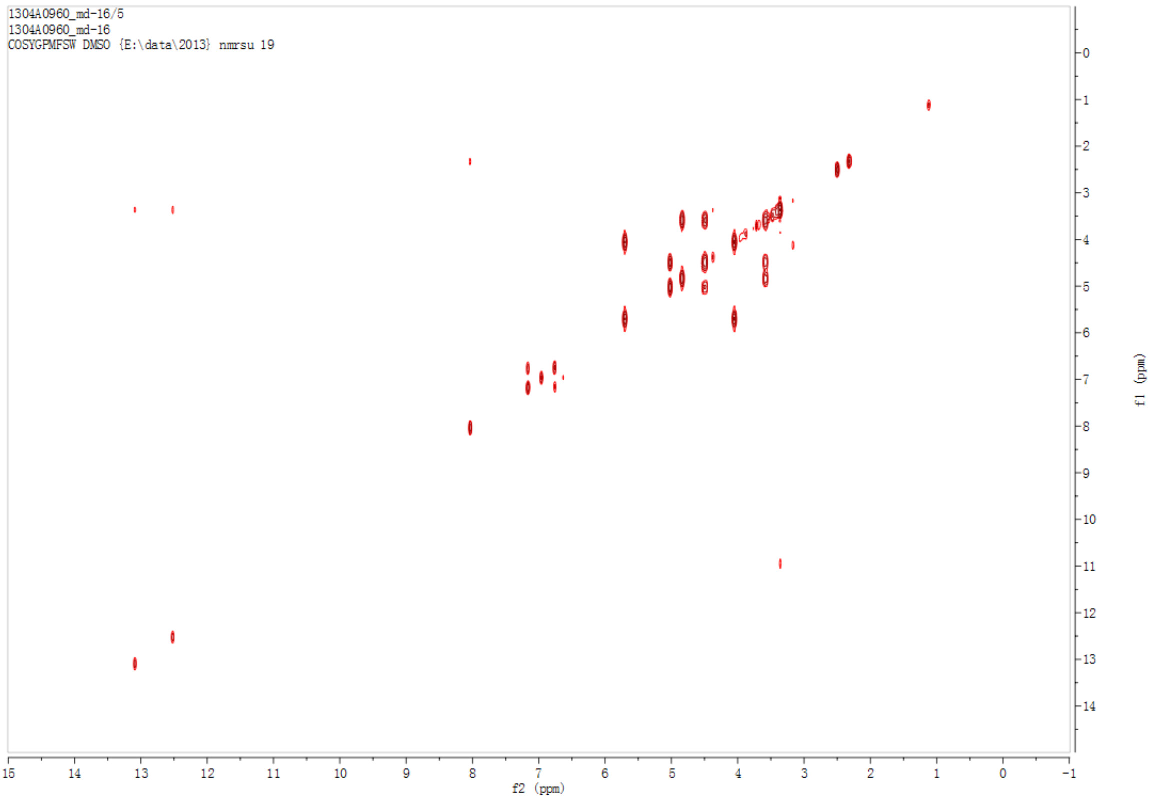

Figure S15. HSQC spectrum of **2** (400/100 MHz, DMSO-d_6).

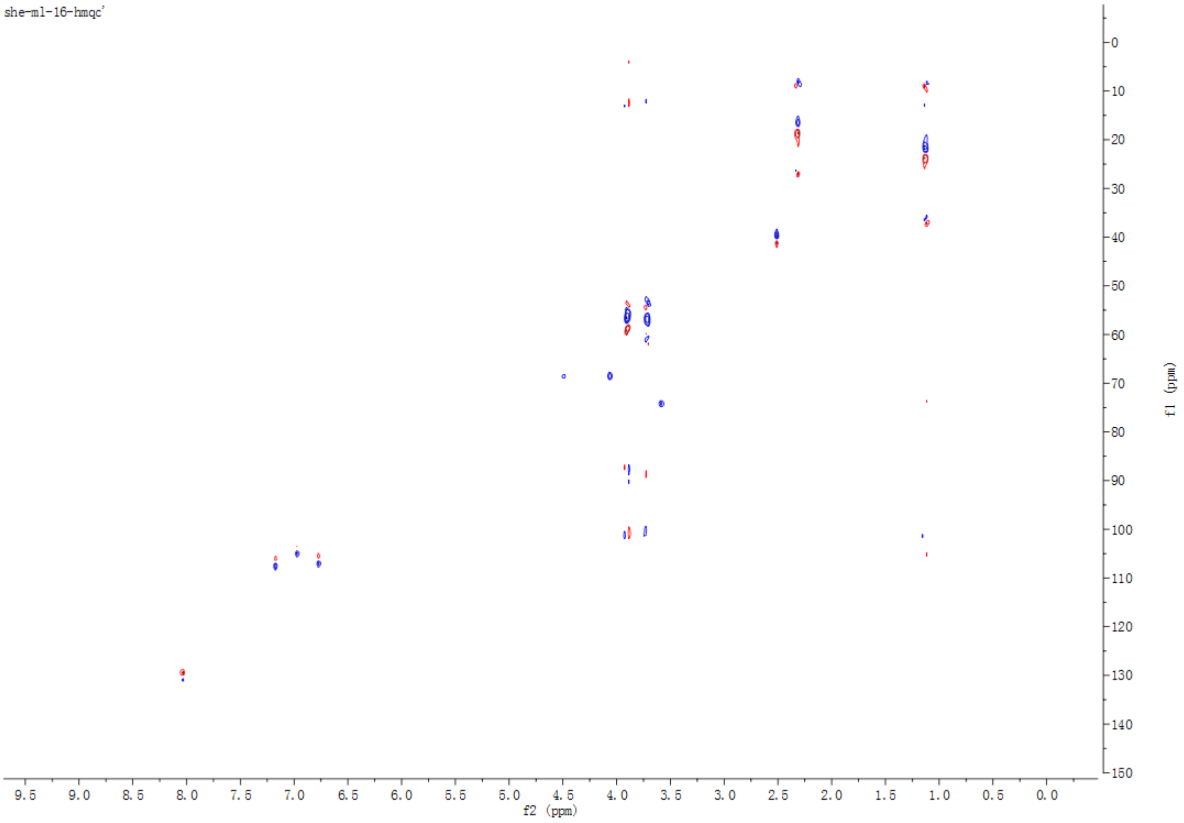

Figure S16. HMBC spectrum of **2** (400/100 MHz, DMSO-d_6).

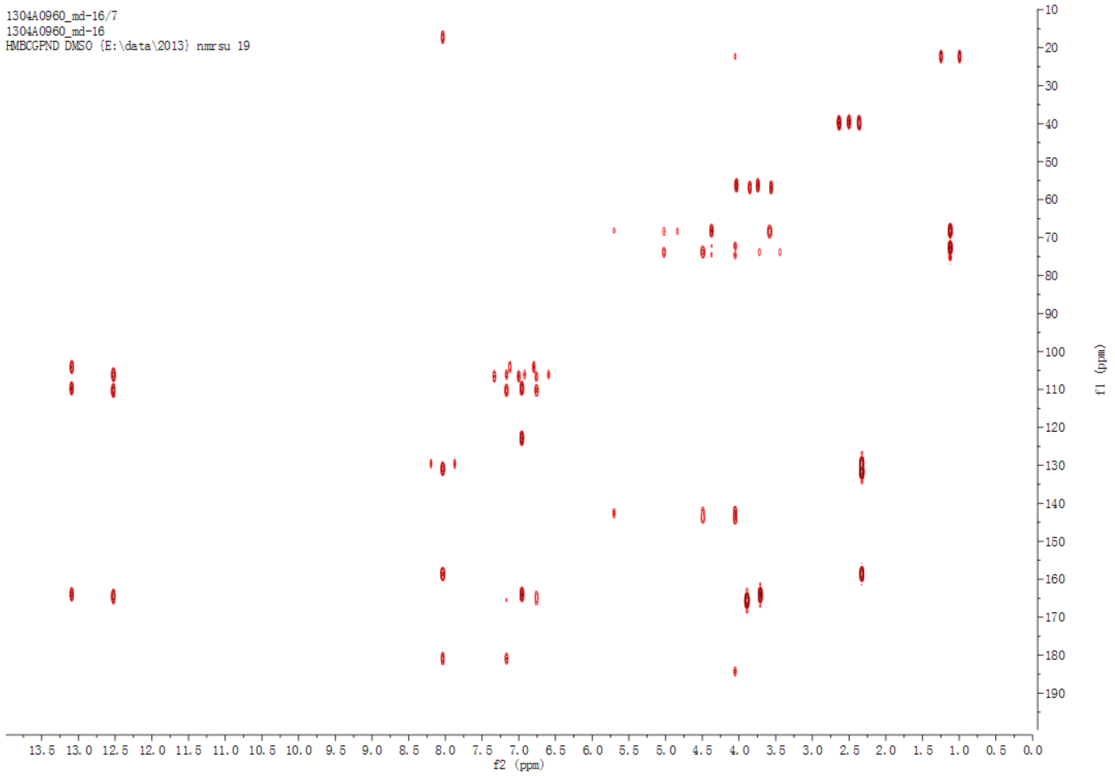

Figure S17. NOESY spectrum of **2** (400 MHz, DMSO-d_6).

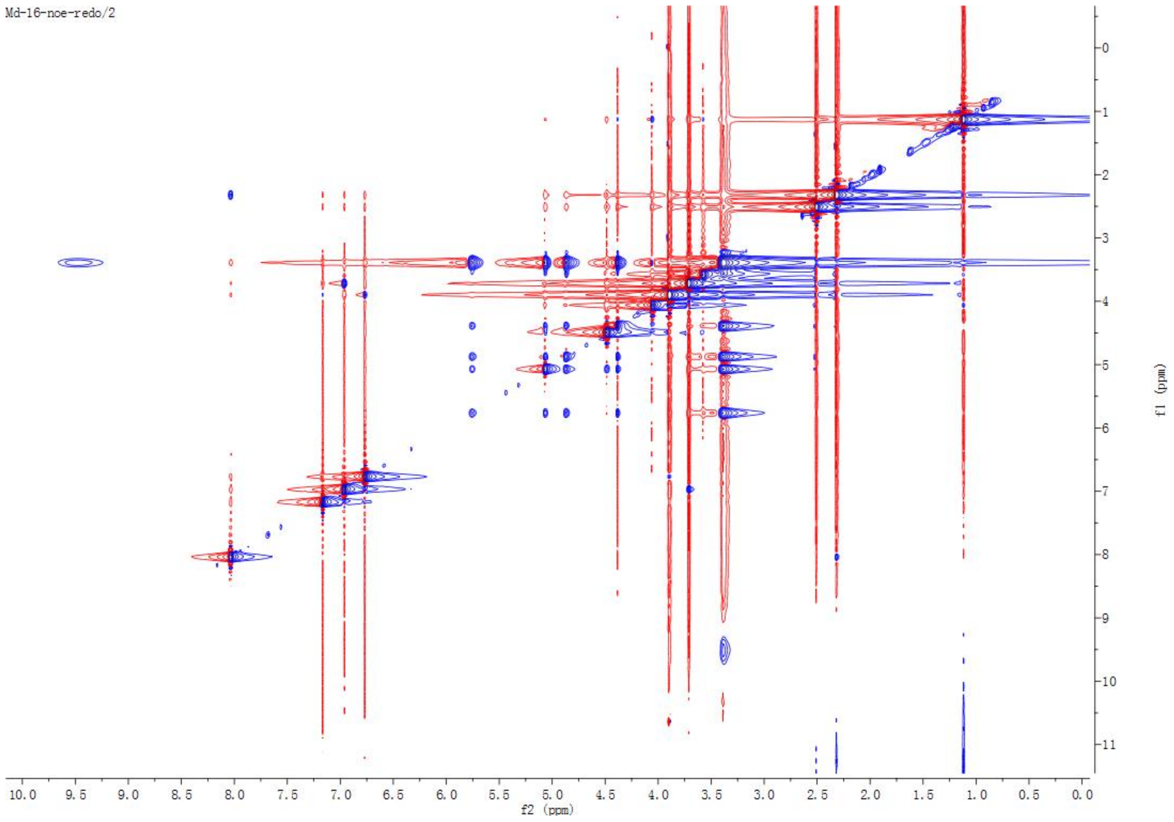

Figure S18. HRESIMS spectrum of **2**.

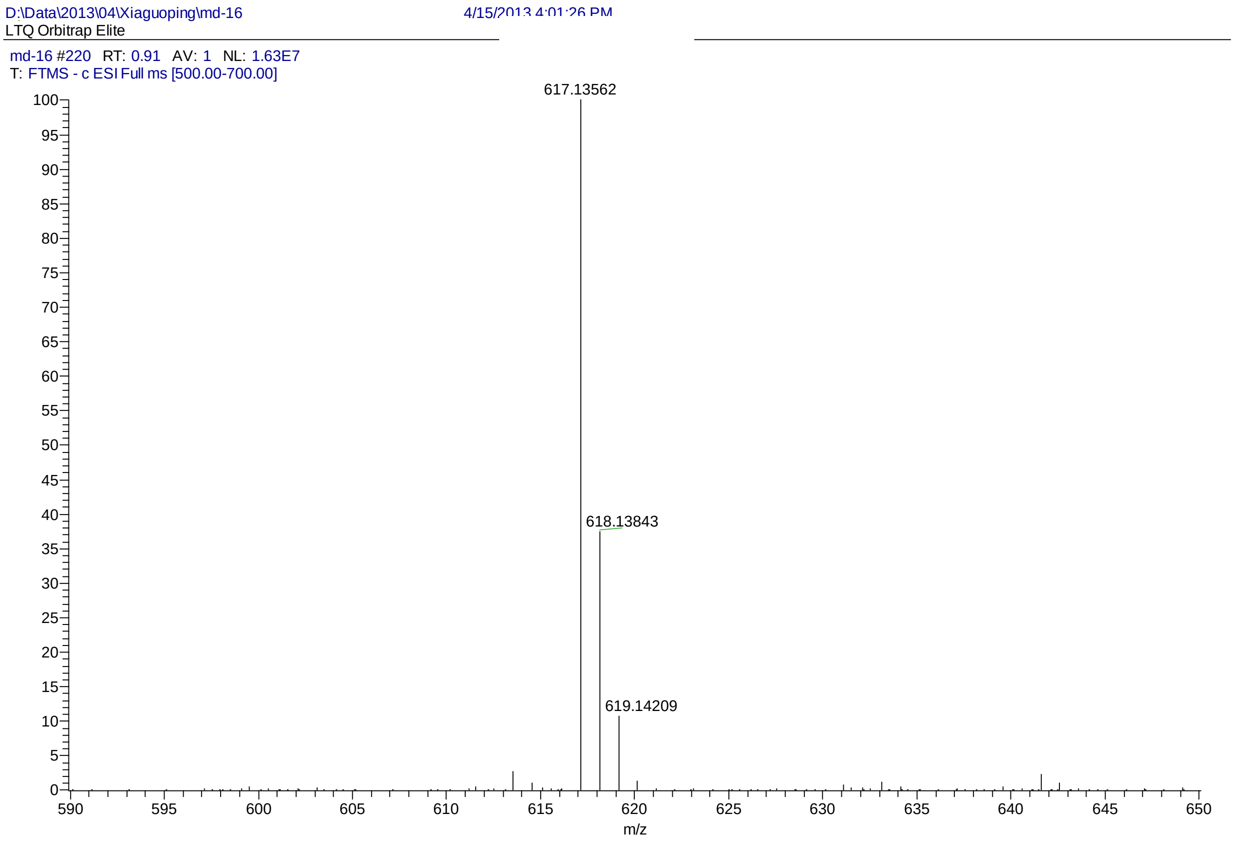

SPECTRUM-simulation:

m/z	Theoretical Mass	Delta (ppm)	RDB Equivalent	Composition
617.13354	617.12897	7.41	20.5	C32 H25 O13

Elements in use: ^{12}C (1~40), ^{16}O (1~20), ^{1}H (1~80).

Figure S19. DFT-optimized low-energy conformations (**2-1**–**2-2**) for a*S*-**2**.

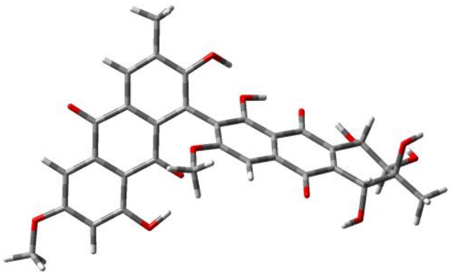

2-1 (ΔE = 0 Kcal/mol)

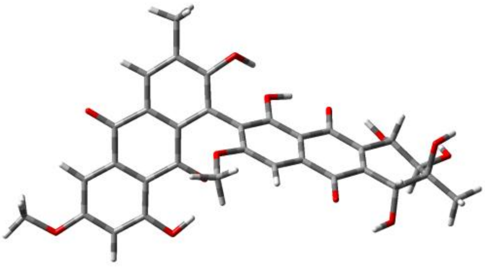

2-2 (ΔE = 0.46 Kcal/mol)

Table S5. TDDFT calculated rotatory strength and oscillator strength results for global minima **2-1**.

1/2[<0|r|b>*<b|rxdel|0> + (<0|rxdel|b>*<b|r|0>)*] Rotatory Strengths (R) in cgs (10**-40 erg-esu-cm/Gauss)					
**State**	**XX**	**YY**	**ZZ**	**R (length)**	**R (au)**
1	48.0493	−90.7944	46.3943	1.2164	0.0026
2	29.6287	19.4358	−12.6477	12.1390	0.0257
3	−25.4646	27.8235	13.6600	5.3396	0.0113
4	−25.3061	−0.3706	56.0028	10.1087	0.0214
5	−40.4521	−9.5944	23.4906	−8.8519	−0.0188
6	−27.0618	−2.4955	−20.3487	−16.6353	−0.0353
7	−37.8221	−4.3778	−21.9333	−21.3777	−0.0453
8	−0.4007	3.3924	−1.9988	0.3310	0.0007
9	226.7585	−23.2613	−127.1331	25.4547	0.0540
10	62.1490	4.5466	−39.8805	8.9384	0.0190
1/2[<0|del|b>*<b|r|0> + (<0|r|b>*<b|del|0>)*] (Au)					
**state**	**X**	**Y**	**Z**	**Dip. S.**	**Osc. (frdel)**
1	−0.0054	−0.0099	−0.0153	0.0305	0.0203
2	−0.0214	−0.0021	−0.0040	0.0275	0.0183
3	−0.1156	−0.0026	−0.0019	0.1201	0.0800
4	−0.0582	0.0000	−0.0028	0.0611	0.0407
5	−0.1576	−0.0003	−0.0008	0.1587	0.1058
6	−0.0024	−0.0001	−0.0002	0.0028	0.0019
7	−0.0313	−0.1039	−0.0032	0.1384	0.0923
8	−0.0007	−0.0005	0.0000	0.0013	0.0008
9	−0.0696	−0.0294	−0.0024	0.1014	0.0676
10	−0.0310	−0.0083	−0.0013	0.0406	0.0271

References

1. Diedrich, C.; Grimme, S. Systematic investigation of modern quantum chemical methods to predict electronic circular dichroism spectra. *J. Phys. Chem. A* **2003**, *107*, 2524–2539.
2. Schellman, J.A. Circular dichroism and optical rotation. *Chem. Rev.* **1975**, *75*, 323–331.
3. Lu, T.; Chen, F.W. Multiwfn: A multifunctional wavefunction analyzer. *J. Comput. Chem.* **2012**, *33*, 580–592.
4. Martin, R.L. Natural transition orbitals. *J. Chem. Phys.* **2003**, *118*, 4775–4777.

© 2014 by the authors; licensee MDPI, Basel, Switzerland. This article is an open access article distributed under the terms and conditions of the Creative Commons Attribution license (http://creativecommons.org/licenses/by/3.0/).
